# QTL and candidate gene mapping for polyphenolic composition in apple fruit

**DOI:** 10.1186/1471-2229-12-12

**Published:** 2012-01-23

**Authors:** David Chagné, Célia Krieger, Maysoon Rassam, Mike Sullivan, Jenny Fraser, Christelle André, Massimo Pindo, Michela Troggio, Susan E Gardiner, Rebecca A Henry, Andrew C Allan, Tony K McGhie, William A Laing

**Affiliations:** 1 The New Zealand Institute for Plant & Food Research Limited (Plant & Food Research), Palmerston North Research Centre, Palmerston North 4442, New Zealand; 2 UMR 1121 Nancy Université-Institut National de la Recherche Agronomique Agronomie Environnement Nancy-Colmar, 2 Avenue de la Forêt de Haye, 54505 Vandoeuvre-lès-Nancy, France; 3 Plant & Food Research, Mount Albert Research Centre, Auckland, New Zealand; 4 Plant & Food Research, Central Otago Research Centre, Clyde, New Zealand; 5 IASMA Research and Innovation Centre, Foundation Edmund Mach, San Michele all'Adige, Trento, Italy; 6 School of Biological Sciences, University of Auckland, Private Bag 92019, Auckland, New Zealand

**Keywords:** *Malus *x *domestica*, polyphenolic, QTL mapping, candidate gene, flavonoid, flavanol, anthocyanin, tannin, metabolomics

## Abstract

**Background:**

The polyphenolic products of the phenylpropanoid pathway, including proanthocyanidins, anthocyanins and flavonols, possess antioxidant properties that may provide health benefits. To investigate the genetic architecture of control of their biosynthesis in apple fruit, various polyphenolic compounds were quantified in progeny from a 'Royal Gala' × 'Braeburn' apple population segregating for antioxidant content, using ultra high performance liquid chromatography of extracts derived from fruit cortex and skin.

**Results:**

Construction of genetic maps for 'Royal Gala' and 'Braeburn' enabled detection of 79 quantitative trait loci (QTL) for content of 17 fruit polyphenolic compounds. Seven QTL clusters were stable across two years of harvest and included QTLs for content of flavanols, flavonols, anthocyanins and hydroxycinnamic acids. Alignment of the parental genetic maps with the apple whole genome sequence *in silico *enabled screening for co-segregation with the QTLs of a range of candidate genes coding for enzymes in the polyphenolic biosynthetic pathway. This co-location was confirmed by genetic mapping of markers derived from the gene sequences. *Leucoanthocyanidin reductase *(*LAR1*) co-located with a QTL cluster for the fruit flavanols catechin, epicatechin, procyanidin dimer and five unknown procyanidin oligomers identified near the top of linkage group (LG) 16, while *hydroxy cinnamate/quinate transferase *(*HCT*/*HQT*) co-located with a QTL for chlorogenic acid concentration mapping near the bottom of LG 17.

**Conclusion:**

We conclude that *LAR1 *and *HCT*/*HQT *are likely to influence the concentration of these compounds in apple fruit and provide useful allele-specific markers for marker assisted selection of trees bearing fruit with healthy attributes.

## Background

Nutritionists have recommended an increased consumption of fruits and vegetables, as sources of dietary compounds such as fibre, micronutrients and antioxidant compounds that are beneficial to human health [1]. Apple (*Malus *x *domestica *Borkh.) is considered to be part of such a healthy diet, being very low in total calories and a good source of dietary fibre (2 g/100 g fresh fruit) [2,3]. While apples are lower in vitamin C than other fruits (5-25 mg/100 g depending on the cultivar [4]), they have very high concentrations of other antioxidant phytochemicals, especially polyphenolic compounds such as quercetin, epicatechin, and procyanidin polymers [2,5,6].

Numerous epidemiological studies have suggested that polyphenolic compounds are involved in the prevention of degenerative diseases such as epithelial (but not hormone-related) cancers and cardiovascular diseases, type-2 diabetes, thrombotic stroke, obesity, neurodegenerative diseases associated with aging and infections [7]. Although polyphenolic compounds have long been studied for their antioxidant properties, which are now well characterized *in vitro*, recent studies have stressed that the mechanisms of biological actions of polyphenols extend beyond their antioxidant properties [8]. It is now believed that polyphenols may exert their beneficial action through the modulation of gene expression and the activity of a wide range of enzymes and cell receptors [9,10]. However, the health effects of dietary polyphenols depend on the amounts consumed and on their bioavailability. Previous studies suggest that the bioavailability of polyphenols is related to their chemical structure [11]. For instance, the nature of the sugar conjugate and the phenolic aglycone are both important for anthocyanin absorption and excretion.

Several thousand compounds having a polyphenolic structure have been identified in higher plants [12,13]. These compounds are secondary metabolites involved in defence against aggression by pathogens or ultraviolet radiation. The most important groups of polyphenols in plants are the flavonoids, phenolic acids, lignans and stilbenes. The flavonoid group can be subdivided into seven subgroups: flavonols, flavones, isoflavones, flavanols, flavanones, anthocyanins and dihydrochalcones of which flavonols, flavanols, anthocyanins and dihydrochalcones are found in apple [14]. The flavonols' main representatives, quercetin and kaempferol, are present in glycosylated forms and the associated sugar moiety is often glucose or rhamnose. Flavanols are not glycosylated, and exist in both the monomer form (catechins) and the polymer form (procyanidins or condensed tannins). Catechin and epicatechin are the main flavanols in fruits and are the building blocks for dimeric, oligomeric and polymeric procyanidins. Anthocyanins are pigments dissolved in the vacuolar sap of usually the epidermal tissues of fruits and exist in a range of chemical forms that are blue, red, purple, pink or colourless according to pH. They are highly unstable as aglycones and in plants are found in glycosylated forms that are stable under light, pH and oxidizing conditions. Cyanidin and pelargonidin are the most common anthocyanins in foods. Finally, dihydrochalcones are a family of the bicyclic flavonoids, defined by the presence of two benzenoid rings joined by a three-carbon bridge. Phloridzin, which belongs to the dihydrochalcone family, is present in apple fruits [14].

Two classes of phenolic acids can be distinguished: derivatives of benzoic acid, and derivatives of cinnamic acid. These acids are found in plants both free and esterified with sugars or other organic acids [15]. Hydroxycinnamic acids are more common than hydroxybenzoic acids, with the main compounds of the hydroxycinnamic acid class being *p*-coumaric, caffeic, and chlorogenic acids. Hydroxybenzoic and hydroxycinnamic acids are also components of complex structures such as hydrolysable tannins (gallotannins and ellagitannins) and lignins, respectively.

Polyphenolic content and identity can vary according to location within the fruit (e.g. skin v. cortex), stage of fruit maturity, location of the fruit within the plant structure and time since harvest. Fruit polyphenolic concentration varies among apple cultivars [2], making this character a potential breeding target. However, current apple breeding programmes emphasize appearance (skin colour, pattern and amount of fruit covered with colour, size and shape of the fruit), eating quality (flavour and texture), and storage ability. Breeding for pest and disease resistances is the second major objective [16]. We are not aware of a breeding programme for apple that includes phytochemical properties and antioxidant content, despite the importance of healthy nutraceutical compounds from apple and other fruit. Consequently genetic mapping studies have focused on fruit quality and disease resistance and only a few have dealt with apple phytochemical content. Quantitative trait loci (QTL) for vitamin C have been identified following analysis of a 'Telamon' × 'Braeburn' segregating population [4] and a major locus for anthocyanin content in apple flesh has been mapped to linkage group (LG) 9 [17]. Expression of this locus has been characterised as being controlled by *MYB10 *[18,19].

This study analyses the genetic control of fruit polyphenolic concentrations using a segregating F_1 _population obtained from a cross between apple cultivars 'Royal Gala' and 'Braeburn', together with information from the apple whole genome sequence (Velasco et al. 2010). Once quantitative trait loci (QTL) were defined, candidate genes coding for enzymes involved in the synthesis of polyphenolic compounds were identified using genome sequencing and confirmed by genetic mapping. On the basis of our results, we suggest that a mutation in *LAR1 *is the probable cause of the variation in the concentration of flavanols in the fruit cortex and skin and that a similar change in *HQT/HCT *possibly causes variation in chlorogenic acid.

## Results

### Analysis of apple polyphenolics by UHPLC in the 'Royal Gala' × 'Braeburn' segregating population

**Figure 1:**
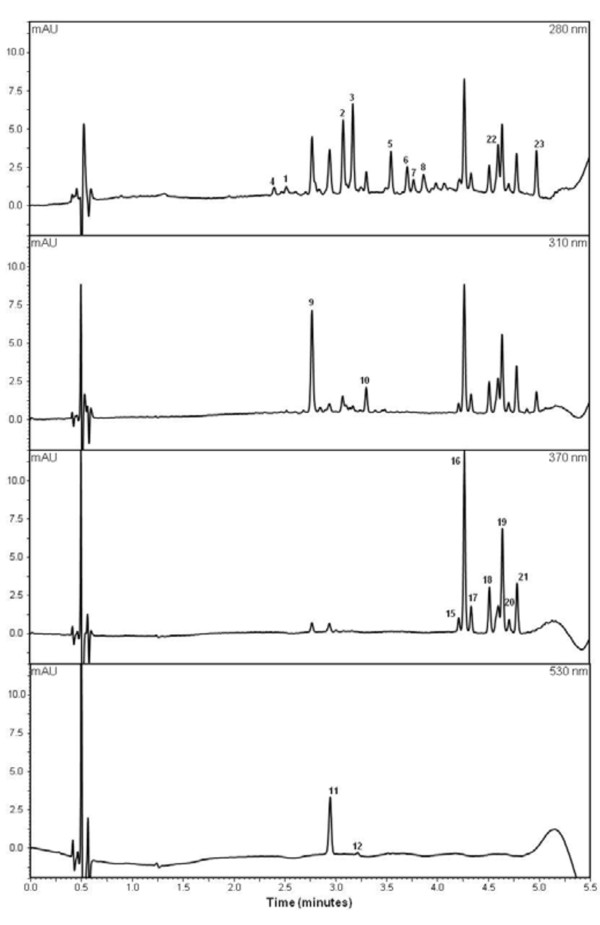
**A representative set of chromatogram traces (280, 310, 370, and 530 nm) typical of apple skin**. This example shows the chromatograms of skin from 'Royal Gala' fruit sampled in 2010. Chromatographic peaks are labelled according to the compound number provided in Table 1. Sample preparation and UHPLC parameters are described, in detail, in the 'Methods' section.

Fruit from a 'Royal Gala' × 'Braeburn' segregating population were analysed using ultra high performance liquid chromatography (UHPLC) in 2008 and 2010 (Figure 1). Twenty-three compounds with variable concentrations were quantified in the apple fruit cortex and skin samples in 2010 and 16 compounds in 2008 (Table 1). The concentration of each compound varied depending on the compound and tissue. The minimum concentration observed for all 23 compounds was below the detection limit of the instrument.

**Table 1:** Range in concentration of 23 polyphenolic compounds isolated from apple fruit skin and cortex of progeny from the 'Royal Gala' × 'Braeburn' segregating population, across two years.

		2008						2010									
#	Compound	Skin			Cortex			Skin					Cortex				
		*Mean*	*Max*.	*Min*.	*Mean*	*Max*.	*Min*.	*Mean*	*Max*.	*Min*.	*BB^A^*	*RG^A^*	*Mean*	*Max*.	*Min*.	*BB^A^*	*RG^A^*
1	(+)-catechin	19.2	131.7	0.0	13.9	135.2	0.0	14.5	172.0	1.0	8.5	15.4	15.4	241.5	2.0	5.0	11.5
2	procyanidin B2	157.2	510.5	0.0	55.0	233.7	0.0	126.7	475.9	4.8	155.7	178.8	31.5	299.2	2.2	48.4	44.9
3	(-)-epicatechin	201.6	709.3	0.0	57.5	277.6	0.0	134.9	533.9	5.8	154.4	209.0	47.7	316.6	2.4	44.1	53.8
4	procyanidin unk1							15.2	113.7	0.5	8.9	22.1	16.7	140.6	1.3	11.8	15.5
5	procyanidin unk2							76.1	275.2	2.5	106.0	105.0	28.4	178.5	1.6	25.7	29.1
6	procyanidin unk3							51.6	169.7	1.7	72.2	60.3	16.1	103.0	1.3	12.8	14.5
7	procyanidin unk4							26.3	89.2	0.8	35.7	28.8	11.5	59.8	1.6	8.2	7.9
8	procyanidin unk5							43.7	142.5	2.0	63.8	54.5	15.6	85.2	1.3	12.6	14.5
9	3-*O*-caffeoylquinic acid	96.4	465.1	0.0	152.6	601.8	0.0	121.2	724.2	8.5	11.9	94.0	177.0	985.9	48.6	99.6	133.5
*10*	*p*-coumaroylquinic acid	28.1	90.8	0.0	56.4	219.9	0.0	32.1	217.4	2.3	22.2	18.5	56.0	343.4	3.0	41.0	38.3
11	cyanidin-3-*O*-galactoside	50.7	324.3	0.0	0.0	0.0	0.0	50.0	242.6	1.2	105.4	45.0	0.0	0.0	0.0	n.d.	n.d.
12	cyanidin-3-*O*-arabinoside	2.8	23.3	0.0	0.0	1.1	0.0	9.7	43.2	1.1	6.2	2.0	0.0	0.0	0.0	n.d.	n.d.
13	cyanidin-unk1							18.2	415.2	0.7	4.8	n.d.	0.0	0.0	0.0	n.d.	n.d.
14	cyanidin-unk2							11.5	25.4	4.8	n.d.	n.d.	0.0	0.0	0.0	n.d.	n.d.
15	quercetin-3-*O*-rutinoside	61.1	228.5	0.0	0.0	0.0	0.0	38.5	217.6	3.7	14.4	17.0	0.0	0.0	0.0	n.d.	n.d.
16	quercetin-3-*O*-galactoside	611.9	1518.2	0.0	0.4	6.5	0.0	389.5	1510.4	16.2	222.9	221.4	0.0	0.0	0.0	n.d.	n.d.
17	quercetin-3-*O*-glucoside	102.1	316.6	0.0	0.1	2.3	0.0	66.3	309.1	14.2	25.0	32.5	0.0	0.0	0.0	n.d.	n.d.
18	quercetin-3-*O*-xyloside	180.9	711.5	0.0	0.1	3.5	0.0	81.6	222.8	29.1	57.1	57.7	0.0	0.0	0.0	n.d.	n.d.
19	quercetin-3-*O*-pyranoarabinoside	201.5	481.0	0.0	0.1	3.1	0.0	156.9	492.8	3.8	85.9	129.4	0.0	0.0	0.0	n.d.	n.d.
20	quercetin-3-*O*-furanoarabinoside	23.2	71.8	0.0	0.0	3.4	0.0	20.4	60.3	4.4	10.7	20.6	0.0	0.0	0.0	n.d.	n.d.
21	quercetin-3-*O*-rhamnoside	98.8	303.8	0.0	0.6	4.8	0.0	80.0	300.1	24.9	53.3	61.4	0.0	0.0	0.0	n.d.	n.d.
22	phloretin-2'-*O*-(2''-*O*-xylosyl)glucoside	44.7	143.2	0.0	9.1	30.6	0.0	53.9	159.3	16.7	43.3	50.7	8.1	42.5	0.7	8.2	8.9
23	phloretin-2'-*O*-glucoside	44.1	167.3	0.0	8.9	23.1	0.0	39.3	112.2	9.1	39.7	38.0	9.2	67.2	1.1	11.0	6.7

The concentrations are expressed in μg/g of fresh weight. Min.: Minimum; Max.: Maximum; RG: 'Royal Gala'; BB: 'Braeburn'.

#### Flavonols

Quercetin glycosides were the only flavonols detected in the apples analysed. Seven main quercetin glycosides were identified: quercetin 3-*O*-rutinoside, quercetin 3-*O*-galactoside, quercetin 3-*O*-glucoside, quercetin 3-*O*-xyloside, quercetin 3-*O*-arabinopyranoside, quercetin 3-*O*-arabinofuranoside, and quercetin 3-*O*-rhamnoside. The most concentrated flavonol was quercetin 3-*O*-galactoside. Quercetin aglycone was detected for two individuals only, one in the fruit skin and another in the cortex. Flavonols predominated in the fruit skin; however, these were virtually absent or not detected in the cortex (Table 1). The correlations between fruit harvested in 2008 and 2010 ranged from *r^2 ^*= 0.14 to *r^2 ^*= 0.38 and there was no correlation for quercetin 3-*O*-xyloside between years. Skin flavonols were highly correlated among themselves and showed medium to high correlations with other polyphenolic compounds present in the skin, such as anthocyanins, flavanols and dihydrochalcones (Additional File 1; Table S1). All flavonol compounds exhibited a skewed phenotypic distribution in the 'Royal Gala' × 'Braeburn' segregating population in both 2008 and 2010 (Additional File 2; Figure S1).

#### Flavanols

Eight flavanol compounds represented the second major class of compounds in both skin and cortex. Three were identified as catechin, procyanidin B2 and epicatechin. Five compounds matched procyanidin spectra and were deemed unknown procyanidin oligomers. Epicatechin was the compound with highest concentrations in both skin and cortex, followed by procyanidin B2. The correlations for flavanol concentration between years were medium (*r^2 ^*= 0.32 for skin catechin) to high (*r^2 ^*= 0.69 for cortex catechin). Flavanols were highly correlated among themselves within the cortex and skin, and between cortex and skin, and with flavonols in the skin. All flavanols showed a skewed distribution in the 'Royal Gala' × 'Braeburn' segregating population, except for cortex procyanidin B2 and the unknown procyanidins 2 and 3, which exhibited bimodal distributions.

#### Anthocyanins

Four cyanidin glycosides were identified: cyanidin 3-*O*-galactoside, cyanidin 3-*O*-arabinoside, and two unknown cyanidins. Anthocyanins predominated in the fruit skin and were virtually absent from the cortex of fruit from the 'Royal Gala' × 'Braeburn' population. The year to year correlations for cyanidin 3-*O*-arabinoside and cyanidin 3-*O*-galactoside were medium high (*r^2 ^*= 0.27 and *r^2 ^*= 0.54, respectively). Cyanidin 3-*O*-galactoside was intermediately correlated with flavanol (*r^2 ^*= 0.45 with quercetin 3-*O*-galactoside) and flavonol compounds in the skin (*r^2 ^*= 0.26 with epicatechin). All anthocyanin compounds exhibited a skewed distribution in the segregating population.

#### Hydroxycinnamic acids

Chlorogenic acid (3-*O*-caffeoyl quinic acid) and *p*-coumaroyl quinic acid were found in both skin and cortex of the apples. Although the total hydroxycinnamic acids were only a small proportion of the total polyphenolic profile of the skin, they represented the major group in the fruit cortex, with chlorogenic acid accounting for nearly 40% of all polyphenolics detected in the cortex. The correlation between years was high for both compounds except for skin *p*-coumaroyl quinic acid (*r^2 ^*= 0.32). Both chlorogenic acid and *p*-coumaroyl quinic acid showed skewed distributions in both skin and cortex in the 'Royal Gala' × 'Braeburn' segregating population.

#### Dihydrochalcones

Phloridzin and phloridzin-xyloside were the only dihydrochalcones found in both skin and cortex of apple. The year to year correlation for both compounds was low to medium (*r^2 ^*= 0.08 to *r^2 ^*= 0.41). Skin phloridzin and phloridzin-xyloside was correlated with both skin flavanol and flavonol compounds. All skin and cortex dihydrochalcones exhibited a skewed distribution in the segregating population.

### Genetic map construction and QTL analysis

A total of 951 single nucleotide polymorphism (SNP) markers originally developed from 'Golden Delicious' to genetically anchor the 'Golden Delicious' apple genome sequence [20] were genotyped in the 590 individuals of the 'Royal Gala' × 'Braeburn' segregating population using 20 SNPlex™ assays. Of these SNP markers, 511 (53.7%) were polymorphic, while 158 (16.6%) failed and 282 (29.6%) were monomorphic. Genetic maps anchored to the apple genome sequence were constructed for both parents using the subset of 170 individuals that was phenotyped for polyphenolic compounds. A subset of 118 SNP markers was used to construct a 'Braeburn' genetic map with as even a marker distribution as possible and favouring backcross type markers (*ab × aa*) above less informative intercross markers (*ab × ab*). This map spanned a cumulative distance of 1,004.8 cM, covered all 17 LGs, had an average of one marker every 8.5 cM and its largest gap was 32.5 cM (Additional File 3; figure S2). One linkage group (LG 7) spanned only 4.1 cM and had only three markers. Ninety-six markers were of the backcross type (*ab × aa*) and 22 were intercross type (*ab × ab*). A subset of 132 SNP markers was used to build the 'Royal Gala' genetic map, using similar distribution and segregation type criteria as for the 'Braeburn' map; 129 of these were of the backcross type and 3 were the less informative intercross type. The cumulative genetic size for the 'Royal Gala' genetic map was 863.9 cM, covering all 17 LGs, with an average of one marker every 6.7 cM and a largest gap of 31.3 cM.

**Figure 2:**
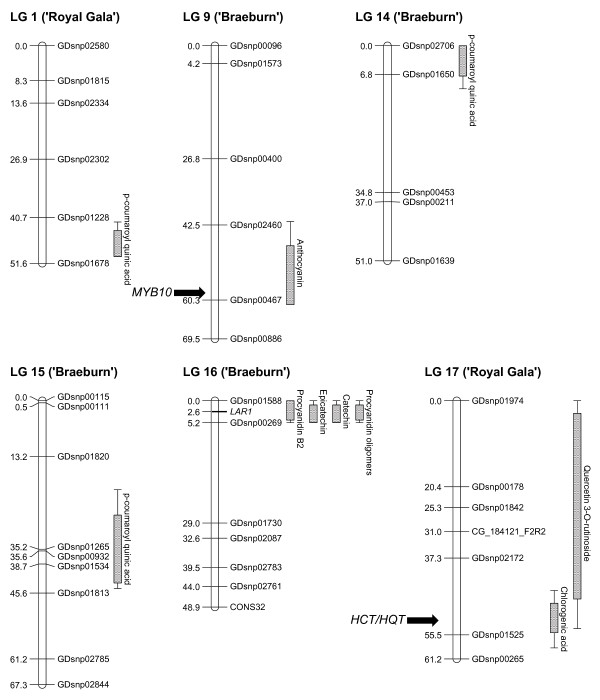
**Stable quantitative trait loci (QTLs) for polyphenolic compounds in apple fruit skin and cortex detected using the 'Royal Gala' × 'Braeburn' genetic map**. Seven clusters detected across 6 linkage groups (LG) were stable using the 2008 and 2010 phenotypic data. QTLs detected in a single year only on LGs 1, 6, 7, 9, 13 and 17 are not presented. The QTL intervals are shown as maximum LOD-1 and maximum LOD-2.

Seventy-nine QTLs for 17 compounds were detected over 9 LGs using multiple QTL (MQM) analysis (Table 2a), with explained genotypic variation ranging from 5% to 71.8%. Four QTLs for Procyanidin B2 and two unknown procyanidin oligomers were detected using the Kruskal-Wallis test (Table 2b). Forty-one and 38 QTLs were detected on the 'Royal Gala' and 'Braeburn' maps, respectively. The largest cluster of QTLs was located at the top of LG 16, where 42 QTLs were detected for flavanol compounds within the same region on both parental maps. Seven clusters were found where QTLs were stable between years and for classes of compounds (Figure 2), including: quercetin 3-*O*-rutinoside in fruit skin for 'Royal Gala' on LG 17; cyanidin 3-*O*-galactoside and cyanidin 3-*O*-arabinoside in fruit skin for both parents on LG 9; all eight measured flavanols in fruit skin and cortex for both parents on LG 16; chlorogenic acid in fruit skin and cortex for 'Royal Gala' on LG 17; *p*-coumaroyl quinic acid in fruit cortex for 'Braeburn' on LG 15; *p*-coumaroyl quinic acid in fruit skin and cortex for 'Royal Gala' on LG 1; *p*-coumaroyl quinic acid in fruit skin and cortex for both parents on LG 14. Other QTLs were detected on LGs 1, 6, 7, 9, 13 and 17 for a range of compounds in individual years, but were not found across years.

**Table 2:** Quantitative Trait Loci (QTLs) detected for 17 polyphenolic compounds in the 'Royal Gala' (RG) and 'Braeburn' (BB) parental genetic maps using a) MQM analysis and b) Kruskal-Wallis test.

Table 2a								
Compound	Tissue	Year	Parent	Linkage group	LOD score		% variation	Marker with highest LOD
Chlorogenic acid	Cortex	2008	BB	13	4.00	**	20.1	GDsnp00101
	Cortex	2008	BB	17	3.67	*	13.2	GDsnp00058
	Cortex	2008	RG	17	8.30	***	30.5	GDsnp01525
	Skin	2008	RG	17	13.98	***	46.5	GDsnp01525
	Skin	2010	RG	17	3.81	**	10.1	GDsnp00178
	Cortex	2010	RG	17	6.25	***	16.3	GDsnp01525
	Skin	2010	RG	17	11.47	***	27.8	GDsnp01525
*p*-coumaroyl quinic acid	Cortex	2008	RG	1	4.44	***	42.4	GDsnp02580
	Skin	2010	RG	1	5.57	**	54.8	GDsnp01678
	Skin	2008	BB	6	3.53	*	12.5	GDsnp00197
	Cortex	2008	BB	14	4.82	**	16.2	GDsnp01733
	Skin	2008	BB	14	4.87	**	17.0	GDsnp01733
	Cortex	2008	RG	14	4.54	***	7.1	GDsnp01727
	Skin	2008	RG	14	4.97	***	19.3	GDsnp01727
	Cortex	2010	BB	14	6.78	***	17.2	GDsnp01767
	Cortex	2010	RG	14	4.14	**	10.9	GDsnp01727
	Cortex	2008	BB	15	3.88	*	12.8	GDsnp01265
	Cortex	2010	BB	15	4.65	***	12.8	GDsnp00932
Cyanidin 3-*O*-arabinoside	Skin	2008	BB	9	4.60	**	18.7	GDsnp00467
	Skin	2008	RG	9	6.26	***	35.4	GDsnp01107
Cyanidin 3-*O*-galactoside	Skin	2008	BB	9	4.53	**	18.7	GDsnp00467
	Skin	2008	RG	9	8.34	***	41.8	GDsnp01880
	Skin	2010	BB	9	8.91	***	23.5	GDsnp00452
	Skin	2010	RG	9	7.30	***	34.1	GDsnp00352
Catechin	Cortex	2008	BB	16	6.44	***	24.5	*LAR1*
	Cortex	2008	RG	16	5.65	***	22.3	*LAR1*
	Cortex	2010	BB	16	18	***	41.1	*LAR1*
	Skin	2010	BB	16	31.26	***	59.9	*LAR1*
	Cortex	2010	RG	16	17.62	***	40.4	*LAR1*
	Skin	2010	RG	16	30.51	***	60.3	*LAR1*
Epicatechin	Cortex	2008	BB	16	18.23	***	55.6	*LAR1*
	Skin	2008	BB	16	16.34	***	51.3	*LAR1*
	Cortex	2008	RG	16	15.53	***	52.0	*LAR1*
	Skin	2008	RG	16	14.89	***	49.3	*LAR1*
	Cortex	2010	BB	16	35.95	***	65.7	*LAR1*
	Skin	2010	BB	16	23.57	***	50.1	*LAR1*
	Cortex	2010	RG	16	32.24	***	65.2	*LAR1*
	Skin	2010	RG	16	21.65	***	48.0	*LAR1*
Unknown procyanidin oligomer 1	Cortex	2010	BB	16	31.82	***	61.2	*LAR1*
	Skin	2010	BB	16	41.28	***	71.8	*LAR1*
	Cortex	2010	RG	16	30.75	***	60.9	*LAR1*
	Skin	2010	RG	16	41.08	***	71.5	*LAR1*
Unknown procyanidin oligomer 2	Skin	2010	BB	6	3.41	*	5.2	GDsnp01996
	Cortex	2010	BB	7	4.30	***	14.3	GDsnp01946
	Skin	2010	BB	16	21.35	***	46.5	*LAR1*
	Skin	2010	RG	16	19.13	***	44.0	*LAR1*
Unknown procyanidin oligomer 3	Cortex	2010	BB	7	3.63	**	13.6	GDsnp01946
	Skin	2010	BB	16	17.15	***	39.5	*LAR1*
	Skin	2010	RG	16	14.97	***	36.4	*LAR1*
Unknown procyanidin oligomer 4	Cortex	2010	BB	7	3.24	*	5.2	GDsnp01946
	Cortex	2010	BB	16	28.38	***	56.6	*LAR1*
	Skin	2010	BB	16	17.33	***	39.8	*LAR1*
	Cortex	2010	RG	16	25.82	***	55.0	*LAR1*
	Skin	2010	RG	16	15.27	***	37.1	*LAR1*
Unknown procyanidin oligomer 5	Cortex	2010	BB	7	3.76	***	5.0	GDsnp01946
	Cortex	2010	BB	14	3.22	**	6.0	GDsnp01639
	Cortex	2010	BB	16	28.3	***	56.6	*LAR1*
	Skin	2010	BB	16	17.93	***	40.9	*LAR1*
	Cortex	2010	RG	16	25.8	***	54.8	*LAR1*
	Skin	2010	RG	16	15.56	***	37.6	*LAR1*
Procyanidin B2	Skin	2008	BB	16	14.14	***	46.5	*LAR1*
	Skin	2008	RG	16	12.84	***	44.5	*LAR1*
	Skin	2010	BB	16	16.6	***	39.6	*LAR1*
	Skin	2010	RG	16	15.59	***	37.6	*LAR1*
Quercetin 3-*O*-arabinofuranoside	Skin	2008	RG	9	3.98	***	20.6	GDsnp00045
Quercetin 3-*O*-glucoside	Skin	2010	RG	1	3.14	*	39.9	GDsnp01678
	Skin	2010	RG	14	3.03	*	26.4	GDsnp01522
Quercetin 3-*O*-rutinoside	Skin	2008	RG	17	3.24	***	12.8	GDsnp00178
	Skin	2010	RG	17	3.17	**	8.4	GDsnp01842
Quercetin 3-*O*-xyloside	Skin	2008	RG	17	3.73	***	14.7	GDsnp02075
Phloridzin-xyloside	Skin	2010	RG	17	3.89	**	10.3	GDsnp00262

**Table 2b**								
Compound	Tissue	Year	Parent	Linkage group	Marker	*K*		

Procyanidin B2	Cortex	2008	BB+RG	16	*LAR1*	43.2		
	Cortex	2010	BB+RG	16	*LAR1*	61.9		
Unknown procyanidin oligomer 2	Cortex	2010	BB+RG	16	*LAR1*	95.3		
Unknown procyanidin oligomer 3	Cortex	2010	BB+RG	16	*LAR1*	83.2		

Significance of the QTL at the genome-wide level was tested using 1,000 permutations: *: 90%, **: 95% and ***: 99%.

### Candidate gene co-location with QTLs

Candidate genes predicted on the basis of involvement in the polyphenolic biosynthetic pathway (Additional File 4; Figure S3) were located bioinformatically on the 'Golden Delicious' whole genome sequence assembly using BLASTN analysis (Table 3).

**Table 3:** Candidate genes for control of fruit polyphenolic content found in the apple gene prediction set (Additional File 2; figure S1).

Symbol	Gene name	Genbank accession	Apple gene prediction	LG	Position (kb)
*PAL*	*phenylalanine ammonia lyase *	ES790093	MDP0000191304	4	8,075
			MDP0000388769	12	15,419
*C4H*	*cinnamate 4-hydroxylase *	EB139247	MDP0000229348	11	5,193
			MDP0000225698	3	5,531
*HCT/HQT*	*hydroxycinnamoyl CoA shikimate/quinate hydroxycinnamoyl transferase*	AM690438	MDP0000264424	9	27,594
		(artichoke HQT1)	MDP0000307780	17	19,929
*C3H*	*p-coumarate 3-hydroxylase *	NP_850337	MDP0000466557	8	35,073
			MDP0000836708	15	53,887
*CHS*	*chalcone synthase *	AB074485	MDP0000686661	9	16,919
			MDP0000686666	9	16,932
			MDP0000302905	14	26,679
*CHI*	*chalcone isomerase *	CN946541	MDP0000759336	14	12,382
			MDP0000682953	1	20,836
			MDP0000205890	11	19,398
*F3H*	*flavanone 3-hydroxylase *	AB074486	MDP0000704377	2	11,905
			MDP0000139343	5	23,797
*F3'H*	*flavanone 3'-hydroxylase *	ACR14867	MDP0000190489	6	26,649
			MDP0000286933	14	31,156
*DFR*	*dihydroflavonol reductase *	AF117268	MDP0000494976	12	26,022
			MDP0000440654	8	4,393
*ANS*	*anthocyanidin synthase (LDOX)*	AF117269	MDP0000360447	6	17,129
*UFGT*	*UDP-glucose flavonoid 3-O-glucosyl transferase *	AF117267	MDP0000376418	9	31,316
			MDP0000706999	7	14,018
*FLS*	*flavonol synthase *	EB137300	MDP0000260404	8	17,498
*LAR*	*leucoanthocyanidin reductase *	AY830131	MDP0000376284	16	1,536
		AY830132	MDP0000140621	13	2,849
*ANR*	*anthocyanidin reductase (Banyuls)*	DQ099803	MDP0000243194	5	2,430
			MDP0000494976	12	26,022
*GT1*	*glucosyl transferase *	EB124403	MDP0000146703	1	14,114
			MDP0000875654	7	19517
*GT2*	*glucosyl transferase *	EB141701	MDP0000316379	14	16,678

The approximate physical position on the apple linkage group (LG) is given in kilobases (kb) from the top of the LG.

Three candidate genes co-located bioinformatically with QTL clusters related to corresponding compound concentrations. A putative *hydroxy cinnamate transferase/hydroxy quinate transferase *(*HCT/HQT*) was located near the bottom of LG 17, where there is a stable chlorogenic acid QTL. The predicted apple protein on LG 17 for HCT and HQT (MDP0000307780) was highly similar to artichoke HCT (86%) and HQT1 (97%) [21], respectively. *Leucoanthocyanidin reductase 1 *(*LAR1*) [22] was positioned at the top of LG 16 and co-located with the cluster of QTLs for flavanols. Five predicted gene models with identical positions had significant sequence similarities with *LAR1 *in this genomic region. PCR primers were developed from genomic sequences for both *LAR1 *and *HCT*/*HQT*, to enable the candidates to be mapped genetically with respect to the respective QTLs. *Anthocyanidin synthase *(*ANS*), located on LG 6 within a QTL from 'Braeburn' for *p*-coumaroyl quinic acid in the fruit skin, was found in 2008 only.

Seven candidate genes were positioned on linkage groups with no associated QTLs: *PAL, F3H, ANR, FLS, C4H, 4CL *and *DFR*. Eight of the candidate genes were positioned on linkage groups where some QTLs were detected; however, most of these candidate genes did not locate within the QTL intervals, or if they co-located there was not an obvious functional biosynthetic pathway correlation between the candidate gene and the compound for which the QTLs were identified.

The high resolution melting (HRM)-based genetic marker for *HCT/HQT *mapped at the bottom of LG 17 of the 'Braeburn' parental map. Location of the candidate gene within the QTL interval was hence determined based on alignment of the 'Braeburn' map with the 'Royal Gala' map (Additional File 3; figure S2).

**Figure 3:**
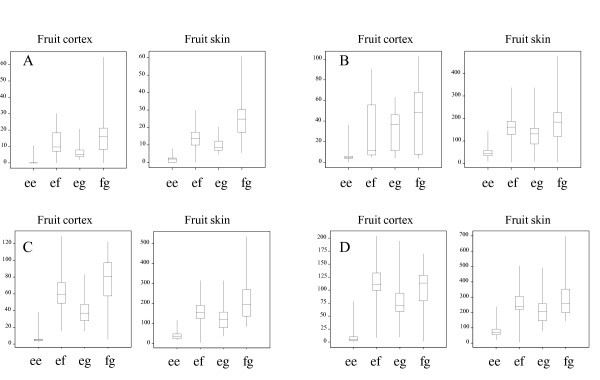
**Phenotypic distribution of four flavanol compounds measured in 2010 in the 'Royal Gala' × 'Braeburn' segregating population based on the *LAR1 *genetic marker genotypes**. A: catechin, B: procyanidin-B2, C: epicatechin and D: procyanidin oligomers. The concentrations are expressed as μg/g of fresh weight.

The HRM-based genetic marker for *LAR1 *that mapped at the top of LG 16 was fully informative, segregating *ef *x *eg*, and it could be employed for QTL detection in both parental maps. The PCR primers for this polymorphic marker were positioned in the fourth intron of *LAR1*. This marker had the highest LOD score for all eight flavanol compounds in both parental maps (Table 2a). MQM analysis with *LAR1 *as a cofactor revealed no other QTL within the LG 16 region or anywhere else in the genome. The *f *and *g *alleles for 'Royal Gala' and 'Braeburn', respectively, were associated with higher concentration of flavanols. The individuals carrying the homozygous *ee *genotype exhibited significantly lower concentrations of flavanol compounds in the fruit skin and no flavanol was detected in the fruit cortex (Figure 3).

A comprehensive search of the 1.2 Mb genomic region spanning the flavanol QTL cluster on LG 16 was performed to ascertain whether candidate genes other than *LAR1 *are also located within this QTL interval. In total, 93 apple predicted genes with supporting cDNA sequences were identified between 0.8 Mb to 2.0 Mb on LG 16 (Additional File 5; figure S4). Of these, 12 had possible regulatory or direct effects on polyphenolic biosynthesis (Table 4) and included transcription factors of the classes zinc-finger C2H2, CONSTANS-like, AP2 bZIP, bHLHs, MYBs and MYB-related.

**Table 4:** Positional candidate genes located in the LG 16 QTL interval for fruit flavanol content detected in the 'Royal Gala' × 'Braeburn' segregating population.

Identifier	Best Arabidopsis match	Possible function	Position (kb)	Gene ID
*C2H2 TF*	AT3G18290	RING finger and CHY zinc finger encoding BRUTUS (BTS), E3 ligase protein with metal ion binding and DNA binding domains, which negatively regulates the response to iron deficiency	1,000	MDP0000283750
*C2H2 TF*	AT1G68360	C2H2 and C2HC zinc finger family	1,020	MPD0000183099
*MdMYB7*	AT1G68320	MYB involved in regulation of phosphate starvation responses and gibberellic acid biosynthesis.	1,070	MDP0000659260
*bHLH*	AT1G25330	bHLH75, BR enhanced	1,080	MDP0000725991
*COL*	AT1G68520	Contans-like light regulation of secondary metabolites	1,220	MDP0000185616
*bZIP*	AT1G68640	HBP-1b(c1), BZIP - PAN is essential for AG activation in early flowering	1,380	MDP0000250967
*MdG2L6*	AT1G25550	Golden-like ARR18 MYB-related	1,440	MDP0000202657
*MdAP2D36 TF*	AT1G68840	Regulator of vac ATPase Rav2 is part of a complex that has been named 'regulator of the (H+)-ATPase of the vacuolar and endosomal membranes' (RAVE)	1,480	MDP0000939633
*LAR1*		MdLAR1	1,540	MDP0000376284
*bHLH*	AT1G68810	Known bHLH, no other function	1,550	MDP0000319726
*bHLH*	AT1G68920	bHLH49 Known bHLH, no other function	1,890	MDP0000154272
*MdbHLH21*	AT1G69010	BES1-INTERACTING MYC-LIKE PROTEIN 2	1,970	MDP0000149222

The approximate physical position on the apple linkage group (LG) is given in kilobases (kb) from the top of the LG calculated using assembled metacontigs of the apple genome sequence [20].

**Figure 4:**
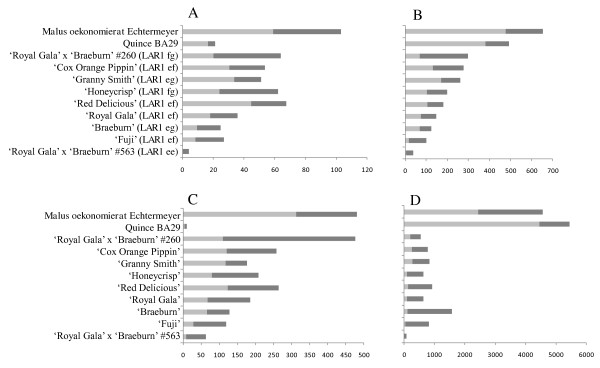
**Flavanol concentration measured in 2010 in fruit skin and cortex of seven commercial apple cultivars compared with quince, crab apple and two seedlings from the 'Royal Gala' × 'Braeburn' segregating population**. The 'Royal Gala' × 'Braeburn' seedlings and cultivars are shown with their *LAR1 *genotype as in Figure 3. A: catechin, B: procyanidin-B2, C: epicatechin and D: procyanidin oligomers. The concentrations are given in μg/g of fresh weight.

We compared the flavanol compounds content of individuals from the 'Royal Gala' × 'Braeburn' segregating population with commercial cultivars, as well as two related species with extreme polyphenolic content: quince and a crab apple (Figure 4). Quince (*Cydonia oblonga*) was at the low extreme of epicatechin content, far below that found for the lowest member of the 'Royal Gala' × 'Braeburn' segregating population, while the highest epicatechin concentration in the 'Royal Gala' × 'Braeburn' progeny was about the same as that in crab apple (*Malus hybrida '*Oekonomierat Echtermeyer*'*). Nevertheless, the apple 'Royal Gala' × 'Braeburn' progeny with the less favourable allele of *LAR1 *(*ee*) was always low.

## Discussion

### Variation in concentration of polyphenolic compounds concentrations in a segregating apple population

The twenty-five fruit polyphenolics compounds analysed by UHPLC varied greatly in concentration among progenies in the 'Royal Gala' × 'Braeburn' segregating population, indicating that there is a serious risk that the process of breeding could rapidly decrease concentrations of nutraceutical compounds positively associated with human health through selection for linked negatively correlated traits. While the parents of our mapping population (both current commercials cultivars) are moderate in their polyphenolic concentrations, apple germplasm exists with far higher concentrations (Figure 4, [2]), indicating the opportunity to raise or lower polyphenolic concentration by breeders. Differences were observed in polyphenolics concentrations in the 'Royal Gala' × 'Braeburn' segregating population between 2008 and 2010. These differences are likely to be due to variations in climatic conditions between both years.

### QTLs for polyphenolic compounds

Our mapping of QTLs for polyphenolic compounds in fruit from the 'Royal Gala' × 'Braeburn' segregating population has added considerably to the body of knowledge concerning the genetic architecture of the control of antioxidant content in apple, previously limited to mapping of QTLs for ascorbate [4], fruit skin anthocyanic colouration [23] and a major locus for fruit cortex anthocyanin content [17]. Seven QTL clusters were stable across two years of harvest and spanned a total of 65 individual QTLs that were highly significant and included loci for content of flavanols, flavonols, anthocyanins and hydroxycinnamic acids. Although the further 14 QTLs putatively controlling concentration for eight polyphenolic compounds detected from either 2008 or 2010 fruit harvests still require verification, their number indicates the complexity of control of biosynthesis in apple fruit of compounds contributing to human health and points towards the influence of environmental as well as genetic factors.

A QTL cluster for skin anthocyanins was detected at the bottom of LG 9, in the region where a major locus for skin colouration was mapped earlier using a sequence characterized amplified region (SCAR) marker [24,25]. It has also been demonstrated that *MYB1 *is involved in skin colouration [23]. Our finding confirms these observations.

A QTL cluster for chlorogenic acid co-located with the *HCT/HQT *gene. Chlorogenic acid is the most concentrated polyphenolic compound in the fruit cortex of the apples from the 'Royal Gala' × 'Braeburn' segregating population. Moreover, chlorogenic acid has a strong antioxidant activity, which makes it of primary importance for the health benefit of apple. *HCT *and *HQT *have been demonstrated to contribute to the synthesis of chlorogenic acid in coffee [26] and artichoke [21,27]. Nevertheless, the *HCT *and *HQT *genes remain to be described in apple and further work is required to confirm the involvement of these genes and their variants, to determine the fruit concentration of chlorogenic acid. This includes developing a *HCT*/*HQT *marker for 'Royal Gala' to confirm that the QTL is strongly linked to this trait and studying these genes' expression during fruit development of cultivars with varying concentrations of hydroxycinnamic acids.

The large effect LG 16 QTL cluster controlling the concentrations of fruit catechin, epicatechin, procyanidin oligomers and procyanidin B2 co-locates with *LAR1*, a candidate gene encoding the enzyme that catalyses the conversion of leucoanthocyanidins into catechin, upstream in the biosynthetic pathway of polymerised procyanidins (Additional File 3; figure S2). Twelve genes coding for transcription factors of several classes that could possibly regulate *LAR1 *are also located within this genomic region. Further work is required to determine whether the effect is through changes in gene regulation or through changes in the biochemical functionality of the enzyme encoded by *LAR1*. To this end, we have initiated a study of the pattern of *LAR1 *expression in extreme phenotypes in the mapping population as well as in a range of apple germplasm accessions. *LAR1 *was demonstrated to be expressed in green and red skin of 'Cripp's Red' during fruit development [22], with higher expression at the early stage of fruit development. However, this study was carried out only in one cultivar. In addition to *LAR1*, mRNA expression of the transcription factors located in the QTL intervals will be carried out in our study. A control of flavanol compounds concentration by LAR1 would contrast with the case of anthocyanin content, where it has been shown that control is exerted at the transcription level [19], through activation of the enzymes of the anthocyanin biosynthesis pathway by *MYB10*, through a WD40-bHLH-MYB protein complex. Our favoured hypothesis is that the mutation is in the promoter region of *LAR1*, in a site recognised by the transcription factors regulating it. We believe that it is likely that the variation is not a complete loss of function of *LAR1*, as tannins have a recognized role in the response of plants to biotic and abiotic stress and although there is another copy of the *LAR *gene (*LAR2*) located on LG13, it not appear co-locate with any flavanol QTL.

While our QTL mapping study does not provide definitive proof that *LAR1 *controls the variation in the concentration of flavanols in the fruit cortex and skin, recent functional evidence using gene silencing of *MdANS *strengthens this hypothesis. ANS uses leucoanthocyanidins in competition with LAR and synthesizes anthocyanidins that are converted into either anthocyanins *via *UFGT or epicatechin *via ANR*. The silencing of *MdANS *suppressed red colouration of a red-leaved apple cultivar and significantly increased the concentrations of other polyphenolic compounds, such as hydroxycinnamic acids, catechin and epicatechin in the transgenic plants [28]. While *ANS *is not involved in the synthesis of hydroxycinnamic acids, we found that it co-locates with a QTL for q-coumaroyl quinic acid. Although this QTL was only found in 2008, our results suggest that *ANS *controls this trait variation. The results of Szankowski and colleagues suggested an alternative pathway for the synthesis of epicatechin by epimerisation or by non-stereospecific depolymerisation of procyanidin oligomers [28]. Our findings that epicatechin and catechin are both positively correlated (0.77 and 0.65 in skin and cortex, respectively), that the QTLs for both compounds co-locate with *LAR1 *and that the alleles of *LAR1 *have the same effect for concentrations of both compounds reinforce the hypothesis by Szankowski and colleagues of an alternative pathway for epicatechin.

The allele-specific genetic marker developed from *LAR1 *is an excellent candidate for use in marker assisted selection for increased flavanol content, as the LG 16 QTL is stable across years and present in parental maps of 'Braeburn' and 'Royal Gala', cultivars which play a significant role in the New Zealand and other apple breeding programmes internationally. Furthermore a similarly located QTL was identified in a segregating population from a 'Prima' × 'Fiesta' cross (S. Khan and H. Schouten, personal communication), indicating that the LG 16 QTL is carried by a range of other commercial apple cultivars that are used as breeding parents. The use of this gene-based marker for selection for fruit flavanol content is particularly important, because phytochemical compounds are not usually measured by breeders when they perform seedling phenotype assessment during the initial steps of selection that involve controlled crosses over one on more generations, but are only evaluated in released commercial cultivars, when it is too late to influence the final breeding outcome. We suggest that the *LAR1 *marker should be screened over the parents for the original cross, as well as progeny from all generations, to ensure that breeding parents at all stages carry either or both favourable alleles of *LAR1*.

In addition, it is useful to investigate the links between flavanol concentration and current breeding traits. While flavanols have been suggested to be beneficial to human health as a source of antioxidants, they are also associated with astringency, which is not preferred by consumers. We are hence planning an organoleptic analysis of fruit from the 'Royal Gala' × 'Braeburn' population to determine whether astringency is correlated with the flavanol concentration. It is known that the LG 16 QTL cluster co-locates with a major locus for fruit acidity (malic acid) identified from a 'Prima' × 'Fiesta' population [25,29] and that acidity is important in determining consumer preferences [30]. In addition, QTLs for loss of fruit firmness during storage and date of budbreak have been mapped to the same region of LG 16 in the 'Royal Gala' × 'Braeburn' population (D. Chagné, unpublished). There is thus a real risk of selecting for a mix of positive and negative traits if the *LAR1 *gene marker is used in isolation. Identification of the functional allele of gene markers for each trait mapping to this region (as we have done for flavanols with *LAR1*) will enable selection of the few genetically elite progeny that carry the most desirable combination of traits from large breeding populations.

## Conclusion

We have detected stable QTLs for polyphenolic antioxidant content in apple and have provided strong evidence that a polymorphism linked to a biosynthetic enzyme for flavanols (*LAR1*) controls the concentration of flavanols in the fruit skin and cortex, and evidence that a polymorphism linked to *HQT/HCT *may control the concentrations of chlorogenic acid. These candidate gene-derived markers have potential to facilitate the development of new apple cultivars bearing fruit with increased concentrations of a range of polyphenolic compounds with human health-benefit.

## Methods

### Plant material, fruit sampling and polyphenolic extraction

In 2005, 590 seedlings from a 'Royal Gala' × 'Braeburn' segregating F_1 _population were grafted on 'M.27' dwarfing rootstocks at the Plant & Food Research Clyde research orchards (Central Otago, New Zealand). Two replicates for each tree were planted at random, 5 × 1.5 m apart in an east-west orientation. The parents 'Royal Gala' and 'Braeburn' were also planted at random in the same block. Trees were regularly irrigated to avoid water deficit, and pests and diseases were controlled by conventional techniques in line with professional practices.

Fruit were picked at maturity based on background colour and blush development followed by a confirmatory starch iodine test between mid February and end of April in 2008 and 2010. A total of 120 and 170 genotypes were sampled in 2008 and 2010, respectively. Six apples were randomly harvested from each tree. The fruit were processed within two to three days of picking. Each apple was cut into four quarters longitudinally through the axis of the core and four thin segments (~5 mm wide at the skin side, tapering to nothing above the core) from were taken from the four quarters of each of the six apples per tree. The skin was removed from each segment to create a separate sample from the cortex. The 24 skin pieces and the 24 cortex pieces from the six apples were bulked separately, frozen immediately in liquid nitrogen and then ground in liquid nitrogen to a fine powder.

Approximately 100 mg of this powder was suspended in 1 ml of extraction solvent (ethanol/water/formic acid, 80:20:1, v/v/v). Each sample was homogenised for 1 minute using a Heidolph^® ^vortex-shaker, Reax 2000 (Heidolph Instruments GmbH, Schwabach, Germany), then stored at 4°C for 24 hours before being centrifuged at 1000 g for 10 min using a Jouan^® ^Hema-C centrifuge (Jouan, Saint Herblain, France). 180 μl of the clear supernatant was transferred to a 96-well plate for analysis by reverse-phase UHPLC. A duplicate was included every ten samples to evaluate the reproducibility.

### UHPLC analysis of fruit polyphenolics

The UHPLC system used to analyse polyphenolics was a Dionex Ultimate^® ^3000 Rapid Separation LC system equipped with a SRD-3400 solvent rack with four degasser channels, HPR-3400RS binary pump, WPS-3000RS thermostated autosampler, TCC-3000RS thermostated column compartment, and DAD-3000RS Diode Array Detector for monitoring at all wavelengths from 190 to 800 nm. The analytical column used was a Kinetex™ C18 1.7 μm, 100 × 2.1 mm (Phenomenex^® ^NZ Ltd., Milford, Auckland City, New Zealand) maintained at 45°C. The injection volume for the extract was 0.5 μl. Linear gradient elution was performed with Solution A (0.5% phosphoric acid in water) and solution B (acetonitrile) delivered at a flow rate of 0.75 ml/min as follows: isocratic elution 5% B, 0-0.5 min; linear gradient from 5% B to 25% B, 0.5-4 min; to 95% B, 4-4.8 min; to 100% B, 4.8-6 min; isocratic elution 100% B, 6-6.5 min; linear gradient to 5% B, 6.5-7 min, to return at the initial conditions before another sample injection at 9 min. Spectral data were collected for the entire run, and the polyphenolic components were quantified by extracting chromatograms at 210, 280, 310, 370 and 530 nm. Catechin, epicatechin and procyanidins were quantified using chromatograms extracted at 210 nm; quercetin, phloridzin and phloridzin-xyloside at 280 nm; *p*-coumaroyl quinic acid at 310 nm; quercetin glycosides and chlorogenic acid at 370 nm; and cyanidin glycosides at 530 nm. Chromatographic data were collected and manipulated using the Chromeleon^® ^Chromatography Management System version 6.8. External calibration curves were constructed for epicatechin, catechin, phloridzin, quercetin, quercetin 3-*O*-rutinoside, cyanidin 3-*O*-glucoside, chlorogenic acid, procyanidin B2, and *p*-coumaroyl quinic acid using standards from Extrasynthese, Genay, France. Components for which standards were not available were quantified using the standard curve of a related compound. All the cyanidin glycosides were quantified using the calibration curve for cyanidin 3-*O*-glucoside. Quercetin glycosides were quantified using the calibration curve for quercetin 3-*O*-rutinoside, phloridzin-xyloside was quantified using the calibration curve for phloridzin, and unknown procyanidins were quantified using the calibration curve for epicatechin.

The initial polyphenolic concentrations were calculated in μg/ml (C_v_). Concentrations (C_w_) in μg/g of fresh weight are obtained using the following formula:

$${\text{C}}_{\text{W}}\left(\mu g/g\;of\;FW\right)=\left[{\text{C}}_{\text{v}}\left(\mu g/ml\right)\times \text{corrected}\;\;\text{volume}\;\left(ml\right)\right]/\text{sample}\;\text{weight}\left(\text{g}\right).$$

Because apples contain on average 85% water, a corrected volume was calculated as:

Correctedvolume(ml)=extractionsolventvolume(ml)+[0.85×sampleweight(g)].

Identification of the polyphenolic compounds in the *Malus *germplasm set was performed using a Dionex Ultimate 3000 system (Sunnyvale, CA) equipped with a diode array detector (DAD). A 5 μL aliquot was injected onto a Dionex C18 Acclaim PolarAdvantage II column (150 × 2.1 mm i.d.; 3 μm particle size) (Sunnyvale, CA). The mobile phases were (A) water with 0.1% formic acid and (B) acetonitrile with 0.1% formic acid. The flow rate was 0.35 mL min^-1^, and the column temperature was 35°C. The 42 min gradient was as follows: 0-5 min, 0-8% B; 5-10 min, 8-15% B; 10-20 min, 15-20% B; 20-27 min, 20% B linear; 27-34 min, 27-100% B; 34-36 min, 100% B linear; 36-42 min, 0% B, re-equilibration time. Simultaneous monitoring was set at 254 nm, 280 nm, and 320 nm, 520 nm for quantification. Polyphenol compounds were identified by their retention time and spectral data compared with standards, and were quantified using five-point calibration curves.

### DNA extraction and SNP marker development

Genomic DNA was extracted from young leaves from the parents and the 590 seedlings of the 'Royal Gala' × 'Braeburn' segregating population using the Qiagen Plant DNeasy Plant Mini kit (Qiagen, Hilden, Germany) following the manufacturer's protocol. Ten nanograms of genomic DNA from each sample were then amplified by whole genome amplification (WGA; [31] using the GenomiPhi V2 DNA Amplification Kit (GE Healthcare, Little Chalfont, Buckinghamshire, United Kingdom). A subset of the 951 single nucleotide polymorphism (SNP) markers used for anchoring the 'Golden Delicious' genome sequence to a genetic map [20] was selected for mapping on the basis of even distribution along the 'Golden Delicious' pseudo-chromosomes. SNPlex™ (Applied Biosystems Inc., Foster City, CA) genotyping assays [32] were carried out using 1 μl (from 45 to 225 ng) of WGA-DNA according to the manufacturer's protocol. Samples were run on a 3730xl DNA Analyzer (Applied Biosystems Inc.) and data were analysed using the Gene Mapper v.4.0 software (Applied Biosystems Inc.). Genotype analysis was performed according to the SNPlex_Rules_3730 method, in accordance with the manufacturer's default settings.

### Genetic map construction and QTL Analysis

Parental genetic maps were constructed using the double pseudo testcross mapping strategy [33]. The linkage analysis and the map construction were performed using JoinMap^® ^v3.0 [34] with a LOD score of 5 for grouping and Kosambi's function for genetic distance calculation. QTL analysis was performed with MapQTL^® ^version 5.0 [35]. The data distribution was verified for each compound before the QTL analysis: non-normal and normal distributions were analysed using a nonparametric (Kruskal-Wallis test) and an interval mapping (IM) analysis, respectively. For the IM analysis, the LOD threshold for significance of a QTL was calculated at the genome level using 1,000 permutations. Only the QTLs with a LOD score significant at greater than 90% genome-wide were retained. The most significant marker for each QTL was then used as a cofactor for a multiple QTL analysis (MQM) for detecting minor QTLs that were hidden by the major QTL in the previous IM analysis.

### Candidate gene detection

Seventeen candidate genes were selected based on their involvement in the polyphenolic biosynthetic pathway (Additional File 3; Figure S2). The sequences of the candidate genes were retrieved from Genbank and a BLAST search performed against the apple gene set predicted from the whole genome sequence of 'Golden Delicious' [20]. The position of each candidate gene was recorded based on its physical position on the apple genome pseudo-chromosomes and then compared *in silico *to the QTL positions. The positions were calculated using assembled metacontigs of the apple genome sequence [20] separated by arbitrary gaps of 200 kb. Candidate genes co-locating with QTLs *in silico *were used to develop polymorphic markers for genetic mapping, to confirm their co-location with QTLs.

### Candidate gene-based marker development and mapping

PCR primers were designed based on the genome assembly contig sequence [20] containing the candidate gene using Primer3 v0.4.0 http://frodo.wi.mit.edu/primer3/. The design criteria were: a final product size ranging from 100 to 150 bp; a primer size ranging from 18 to 27 bp (optimum: 20 bp); a primer melting temperature ranging from 57°C to 63°C (optimum: 60°C); and a percentage of G and C bases ranging from 40% to 55%. The self complementarity and the 3' self complementarity were set to a maximum of 4 and 1, respectively, in order to prevent the formation of primer dimers during PCR. The primers obtained (LAR1_MDC000496.351_F2: TGGTTCCCTGTATCCAAGTTTT, LAR1_MDC000496.351_R2: ACCTGAGATCGGTGTCCTTC, HCT_F2: TGGATGTATAGGTTAGAGAATGTGG and HCT_R2: GCCATTGCTAGATTGACTTTTC) were submitted to a BLASTN analysis against the apple genome assembly to guard against annealing to multiple locations.

The high resolution melting (HRM) technique performed on a LightCycler^® ^480 (Roche) was used to detect sequence polymorphisms [36]. Polymorphic markers were screened over the 'Royal Gala' × 'Braeburn' segregating population and used to construct a new genetic map using the same parameters described earlier.

## Authors' contributions

DC and CK analyzed the chemical datasets, constructed genetic maps, performed statistical and QTL analysis, developed markers for candidate genes and wrote the manuscript. JF collected fruit samples in the field. MR, WAL, CA and MS processed the fruit. MP and MT developed and screened SNP markers in the segregating population. CA, RAH and ACA identified candidate genes in the apple genome sequence. TKM and SEG provided expertise on chemical analysis and genetic mapping, respectively. ACA, DC and WAL designed the experiments edited the manuscript and were project co-leaders. All authors read and approved the final manuscript.

## Supplementary Material

Additional File 1 **table S1: Correlation matrix (*r^2^*) for the concentration of 23 polyphenolic compounds measured in the fruit cortex and skin of the 'Royal Gala' × 'Braeburn' segregating population in 2010**. Click here for file

Additional File 2 **figure S1: Phenotypic districtubtion of polyphenolics compounds detected in fruit skin and cortex of the 'Royal Gala' × 'Braeburn' segregating population in 2008 and 2010**. Click here for file

Additional File 3 **figure S2: Framework genetic map of 'Royal Gala' and 'Braeburn' used for QTL analysis of polyphenolic compounds in apple fruit skin and cortex**. Click here for file

Additional File 4 **figure S3: A simplified schematic of the polyphenolics synthesis pathway. Gene symbols are identified in Table **3. Click here for file

Additional File 5 **figure S4: Apple gene predictions found in the QTL interval for flavanol concentration**. The figure is depicted as a set of GBrowser tracks showing the position on LG 16 (top track), close up position (second and third tracks), apple genome assembly contigs (fourth track), IASMA predicted apple gene set and apple cDNA sequences (fifth track; *Malus *Bioview™) [37] mapping to the region (positioned using *GMAP*). Gene names are indicated where known. Click here for file
