# Supplementary material for: QTL and candidate gene mapping for polyphenolic composition in apple fruit
Source: BMC Plant Biol. 2012 Jan 23;12:12. doi: 10.1186/1471-2229-12-12 (PMC3285079; doi:10.1186/1471-2229-12-12)

3-*O*-caffeoylquinic acid

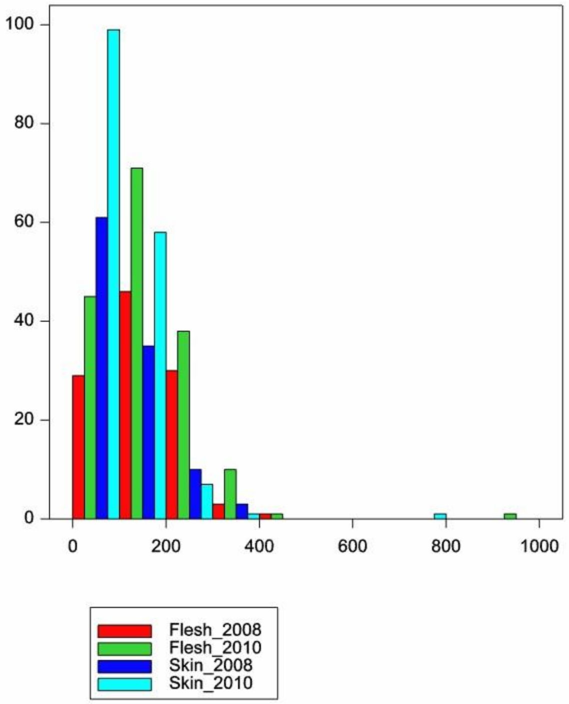

p-coumaroylquinic acid

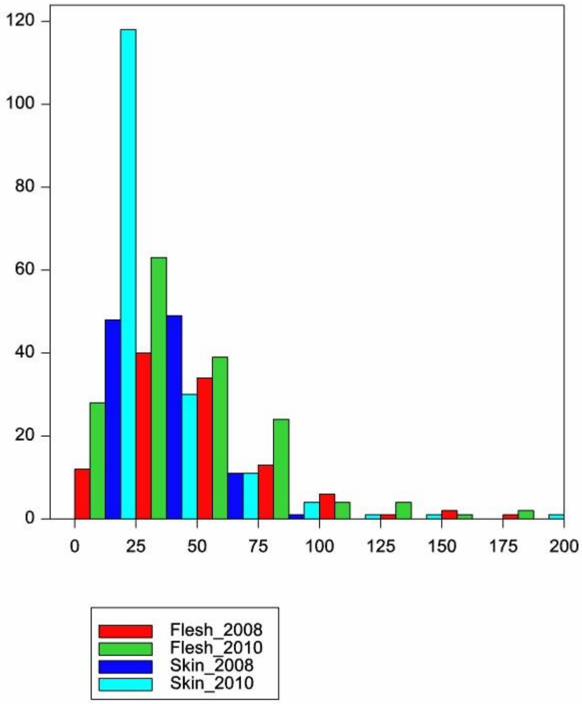

(+)-catechin

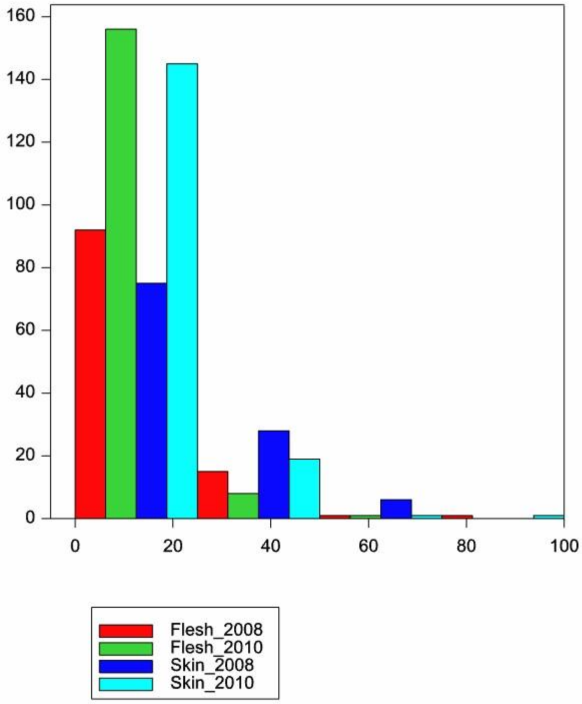

(-)-epicatechin

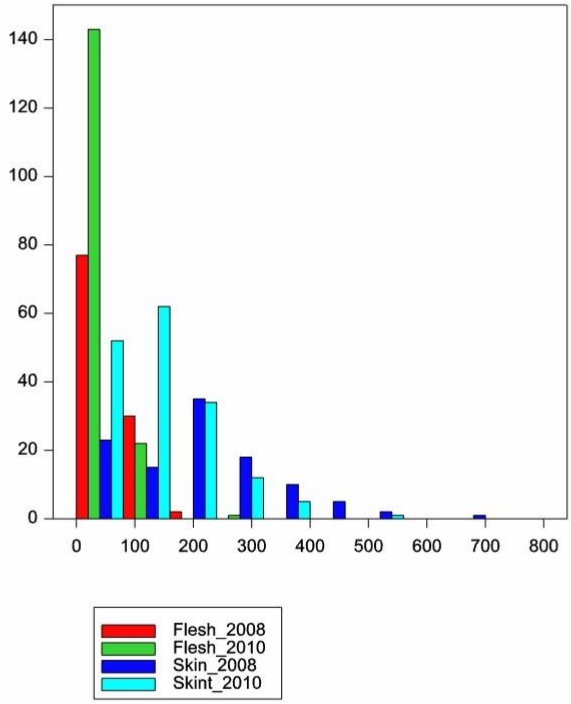

procyanidin unk1

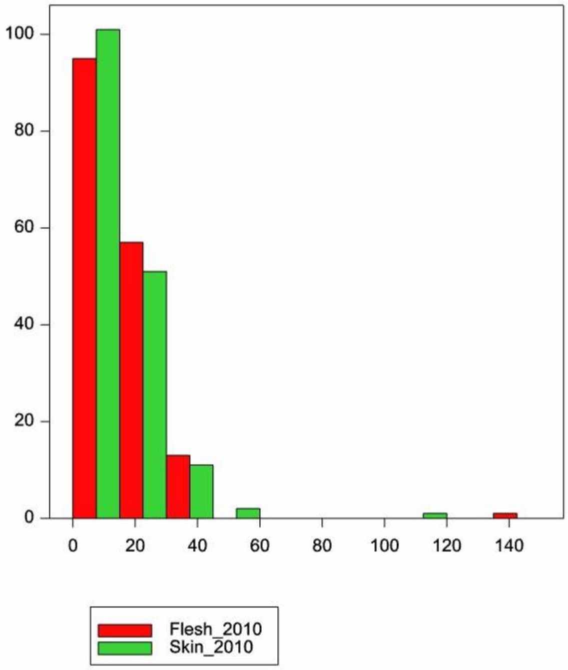

procyanidin unk2

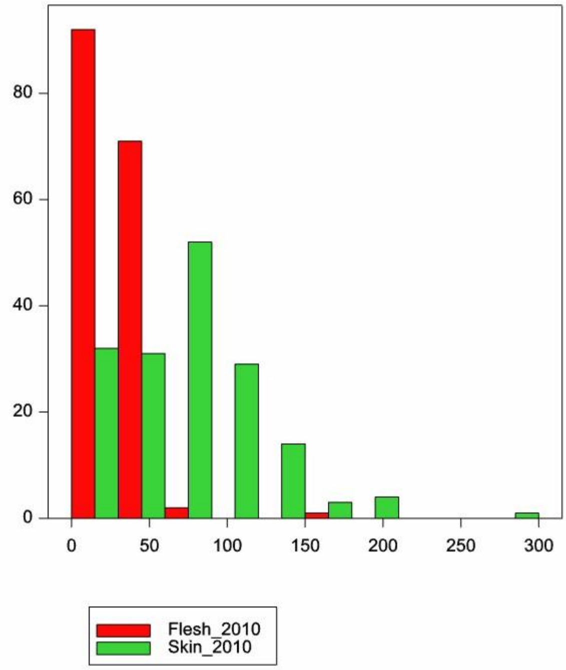

procyanidin unk3

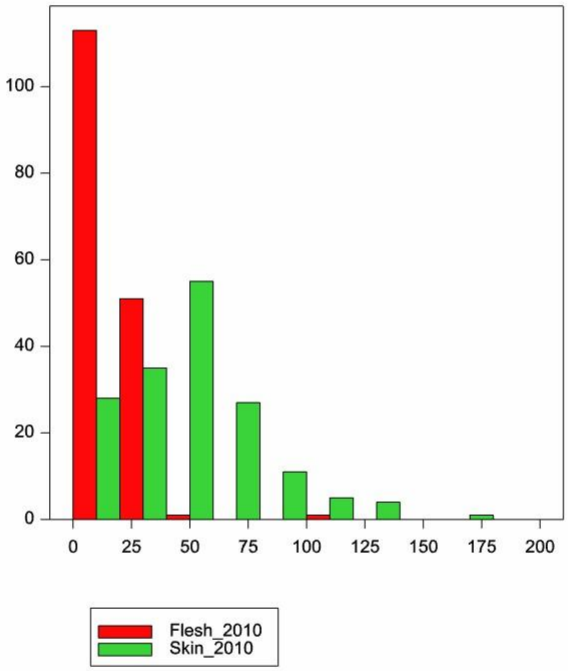

procyanidin unk4

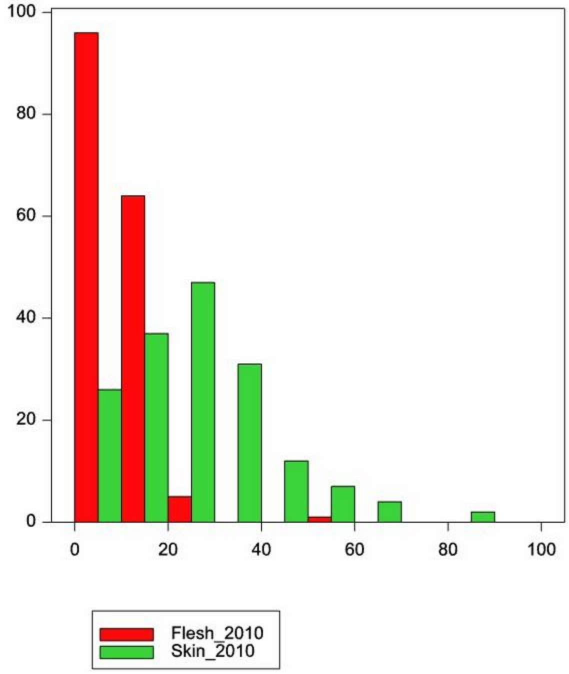

procyanidin unk5

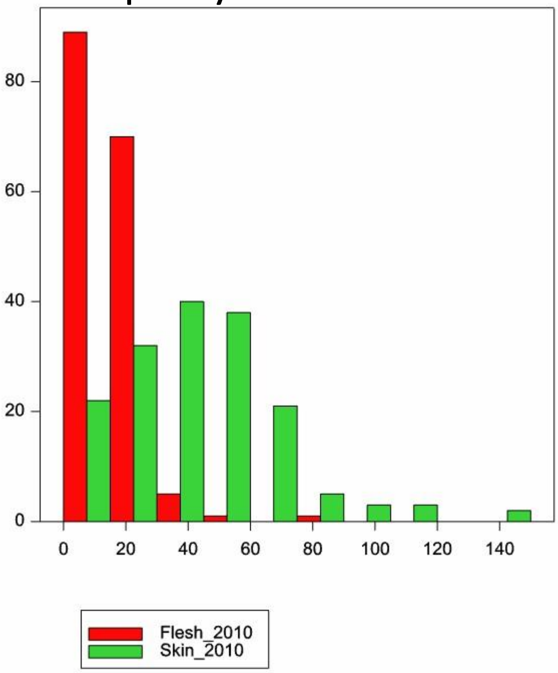

procyanidin B2

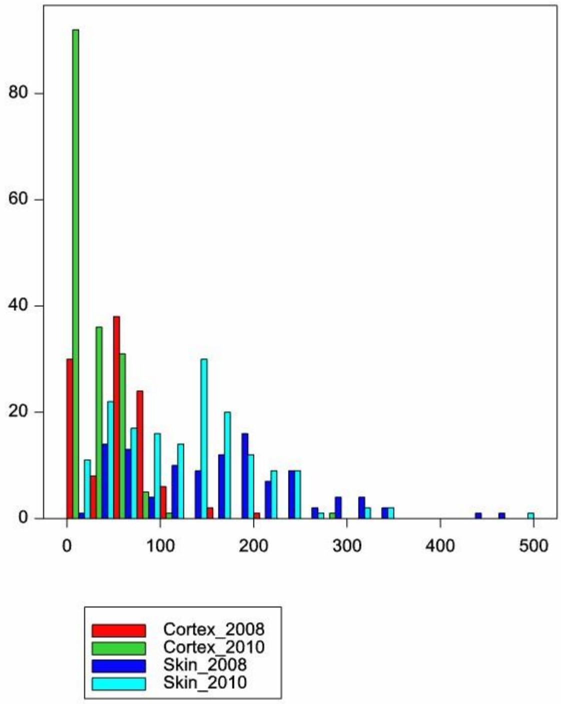

phloretin-2'-*O*-glucoside

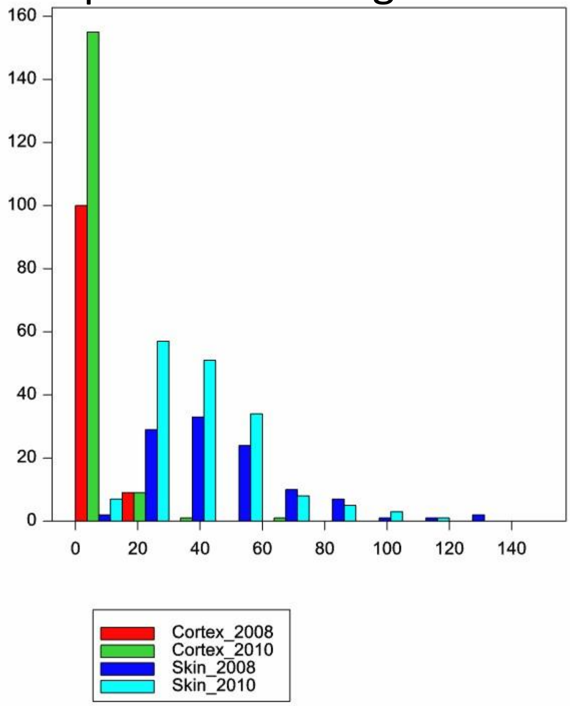

phloretin-2'-*O*-(2''-*O*-xylosyl)glucoside

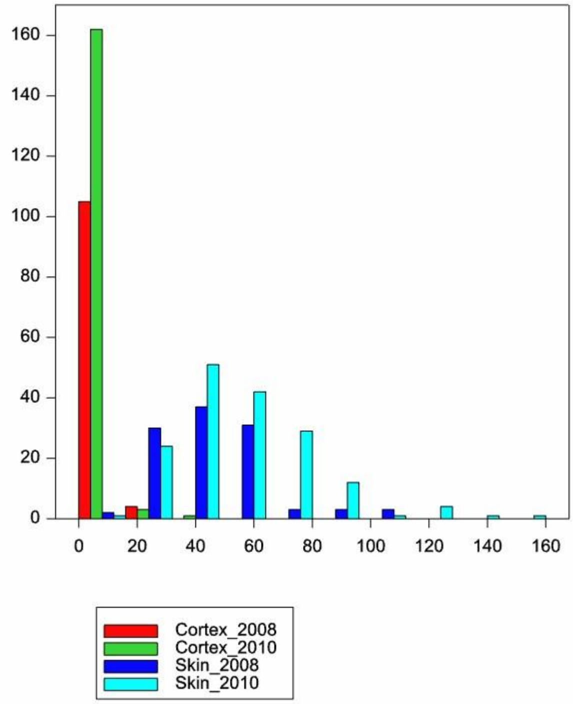

cyanidin-3-*O*-arabinoside

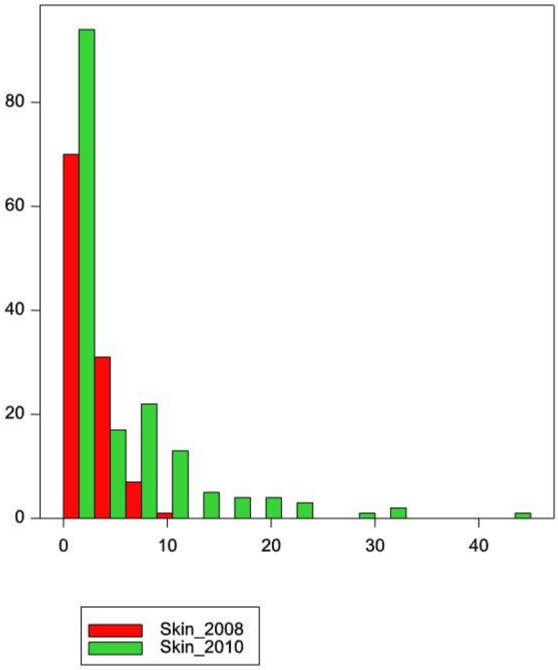

cyanidin-3-*O*-galactoside

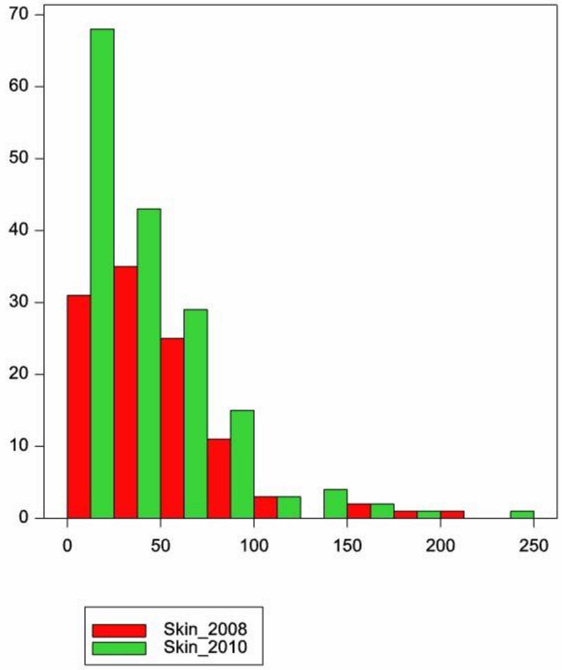

quercetin-3-*O*-galactoside

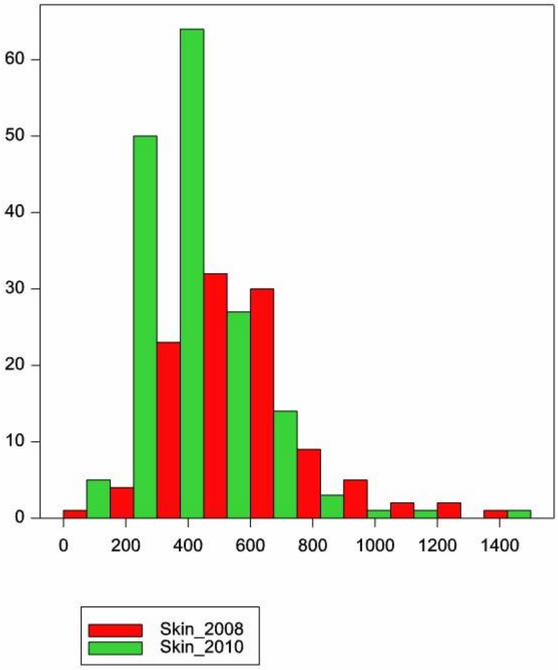

quercetin-3-*O*-glucoside

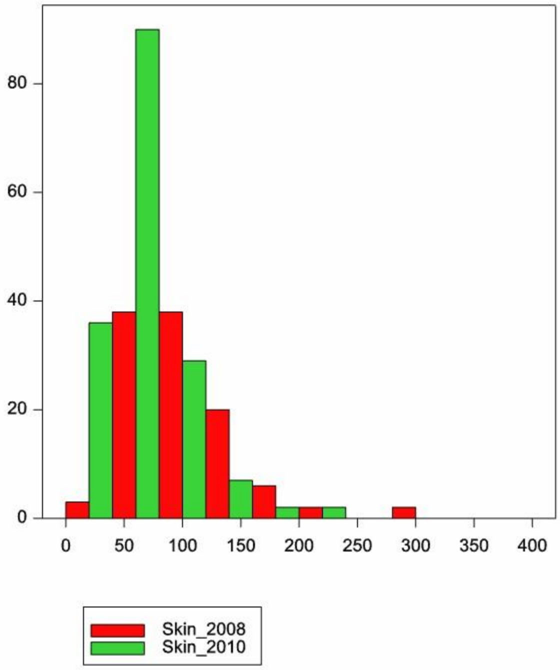

quercetin-3-*O*-rhamnoside

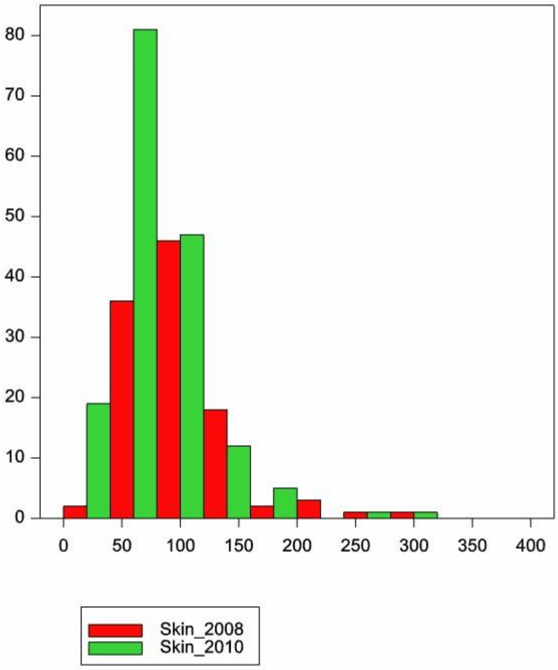

quercetin-3-*O*-rutinoside

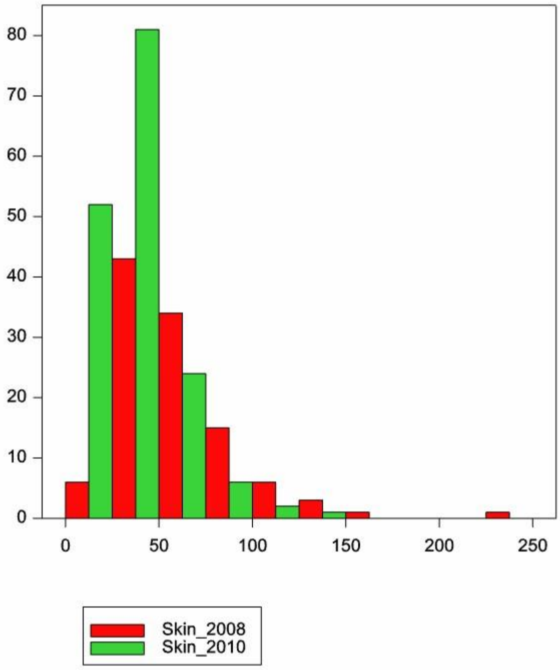

quercetin-3-*O*-xyloside

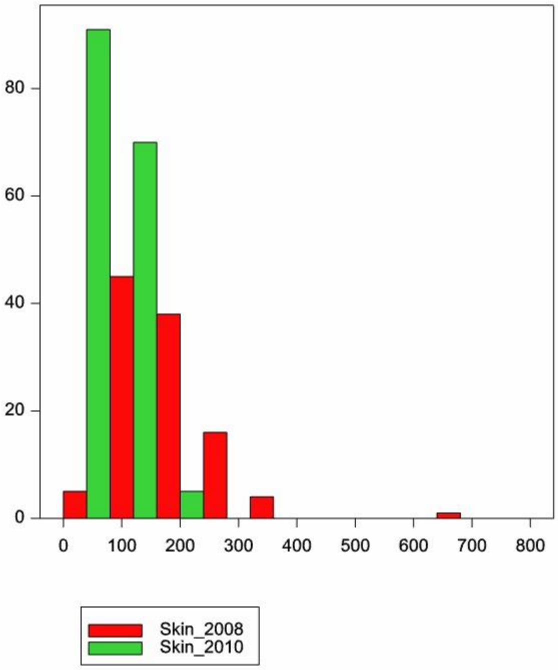

Supplement: Additional File 2 — figure S1: Phenotypic districtubtion of polyphenolics compounds detected in fruit skin and cortex of the 'Royal Gala' × 'Braeburn' segregating population in 2008 and 2010. [file 1471-2229-12-12-S2.PDF]
