# Supplementary material for: QTL and candidate gene mapping for polyphenolic composition in apple fruit
Source: BMC Plant Biol. 2012 Jan 23;12:12. doi: 10.1186/1471-2229-12-12 (PMC3285079; doi:10.1186/1471-2229-12-12)
Supplement: Additional File 3 — figure S2: Framework genetic map of 'Royal Gala' and 'Braeburn' used for QTL analysis of polyphenolic compounds in apple fruit skin and cortex. [file 1471-2229-12-12-S3.PDF]

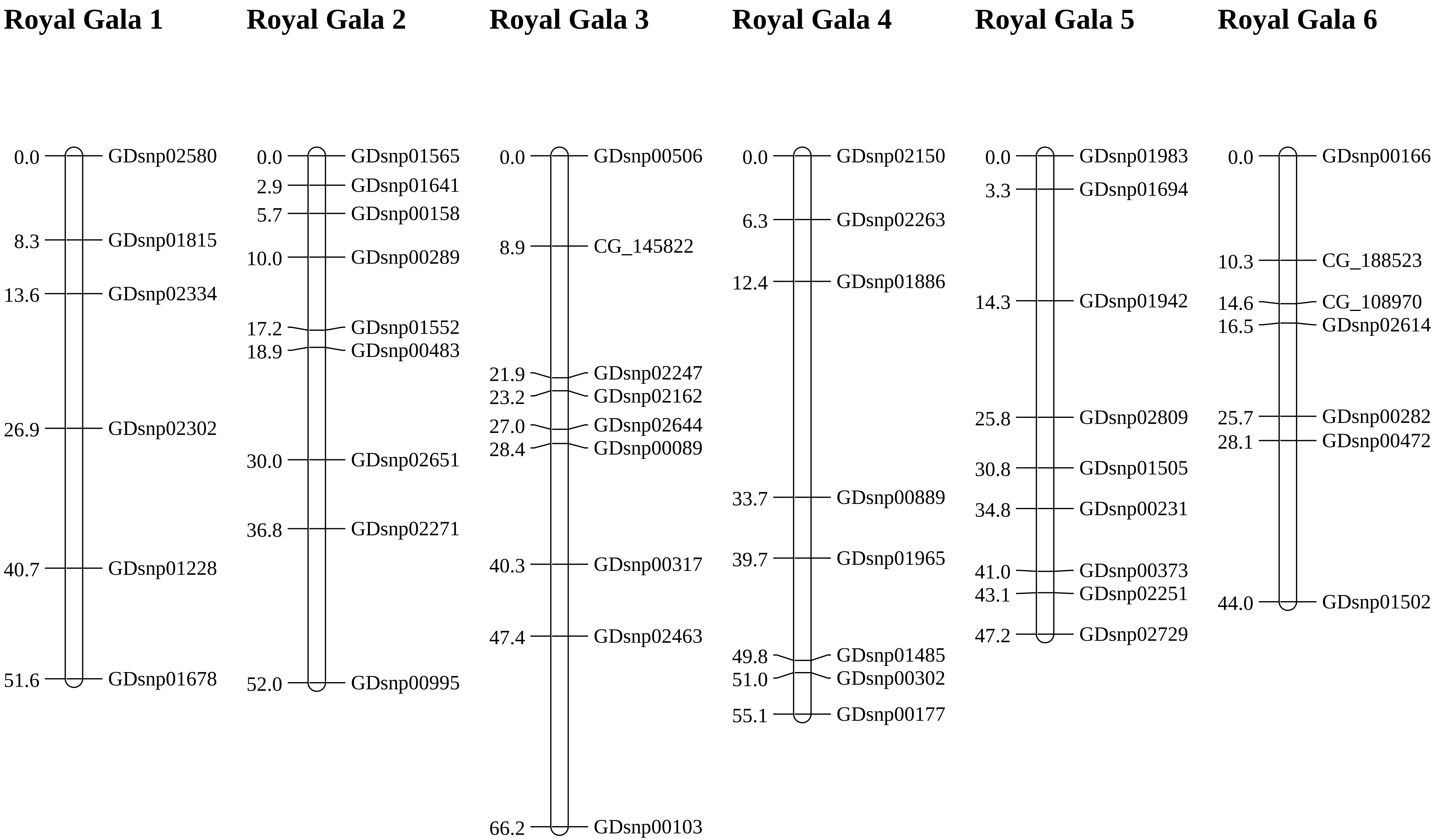
Royal Gala 1
0.0 GDsnp02580
8.3 GDsnp01815
13.6 GDsnp02334
26.9 GDsnp02302
40.7 GDsnp01228
51.6 GDsnp01678
Royal Gala 2
0.0 GDsnp01565
2.9 GDsnp01641
5.7 GDsnp00158
10.0 GDsnp00289
17.2 GDsnp01552
18.9 GDsnp00483
30.0 GDsnp02651
36.8 GDsnp02271
52.0 GDsnp00995
Royal Gala 3
0.0 GDsnp00506
8.9 CG_145822
21.9 GDsnp02247
23.2 GDsnp02162
27.0 GDsnp02644
28.4 GDsnp00089
40.3 GDsnp00317
47.4 GDsnp02463
66.2 GDsnp00103
Royal Gala 4
0.0 GDsnp02150
6.3 GDsnp02263
12.4 GDsnp01886
33.7 GDsnp00889
39.7 GDsnp01965
49.8 GDsnp01485
51.0 GDsnp00302
55.1 GDsnp00177
Royal Gala 5
0.0 GDsnp01983
3.3 GDsnp01694
14.3 GDsnp01942
25.8 GDsnp02809
30.8 GDsnp01505
34.8 GDsnp00231
41.0 GDsnp00373
43.1 GDsnp02251
47.2 GDsnp02729
Royal Gala 6
0.0 GDsnp00166
10.3 CG_188523
14.6 CG_108970
16.5 GDsnp02614
25.7 GDsnp00282
28.1 GDsnp00472
44.0 GDsnp01502

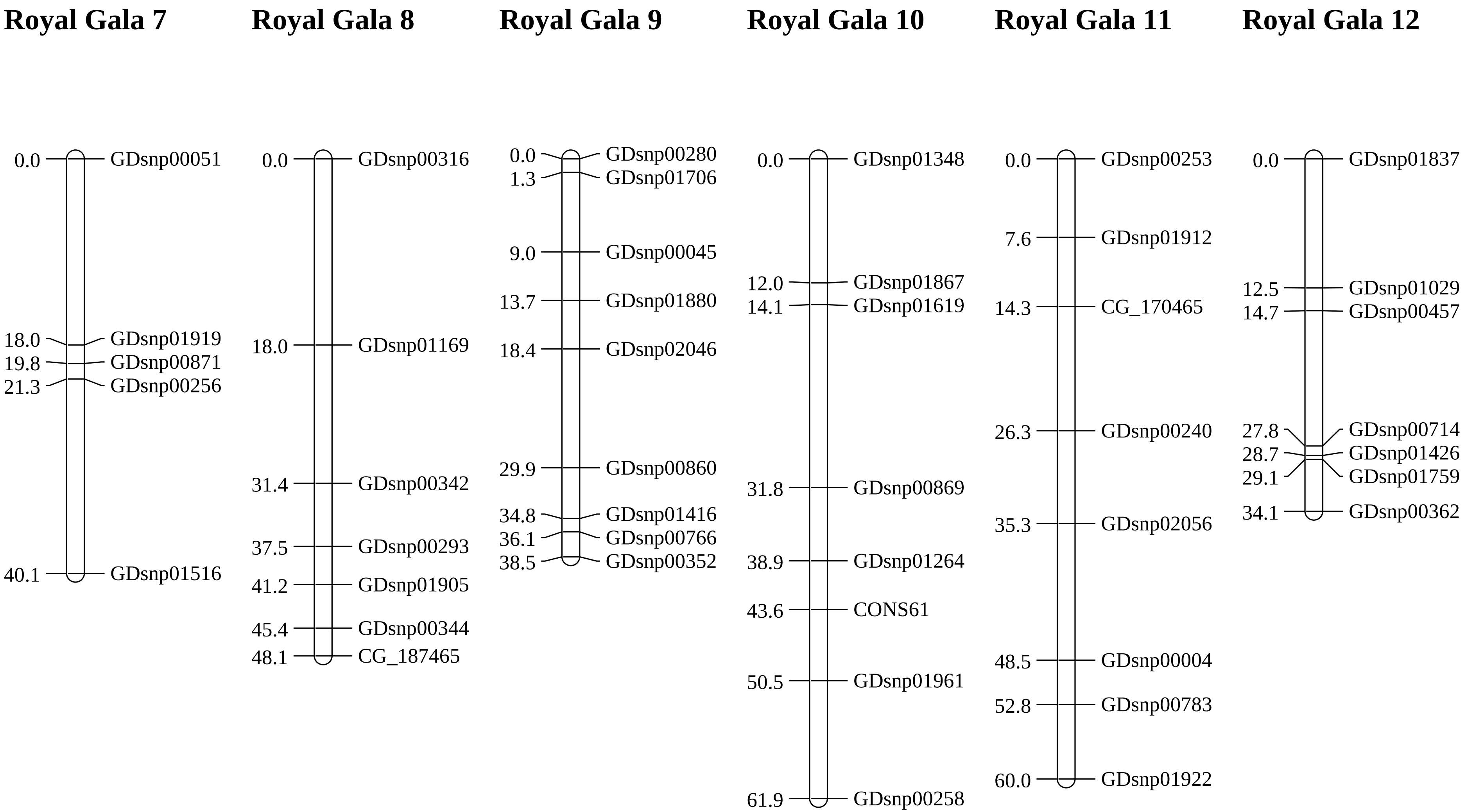

Royal Gala 7
0.0 GDsnp00051
18.0 GDsnp01919
19.8 GDsnp00871
21.3 GDsnp00256
40.1 GDsnp01516
Royal Gala 8
0.0 GDsnp00316
18.0 GDsnp01169
31.4 GDsnp00342
37.5 GDsnp00293
41.2 GDsnp01905
45.4 GDsnp00344
48.1 CG_187465
Royal Gala 9
0.0 GDsnp00280
1.3 GDsnp01706
9.0 GDsnp00045
13.7 GDsnp01880
18.4 GDsnp02046
29.9 GDsnp00860
34.8 GDsnp01416
36.1 GDsnp00766
38.5 GDsnp00352
Royal Gala 10
0.0 GDsnp01348
12.0 GDsnp01867
14.1 GDsnp01619
31.8 GDsnp00869
38.9 GDsnp01264
43.6 CONS61
50.5 GDsnp01961
61.9 GDsnp00258
Royal Gala 11
0.0 GDsnp00253
7.6 GDsnp01912
14.3 CG_170465
26.3 GDsnp00240
35.3 GDsnp02056
48.5 GDsnp00004
52.8 GDsnp00783
60.0 GDsnp01922
Royal Gala 12
0.0 GDsnp01837
12.5 GDsnp01029
14.7 GDsnp00457
27.8 GDsnp00714
28.7 GDsnp01426
29.1 GDsnp01759
34.1 GDsnp00362

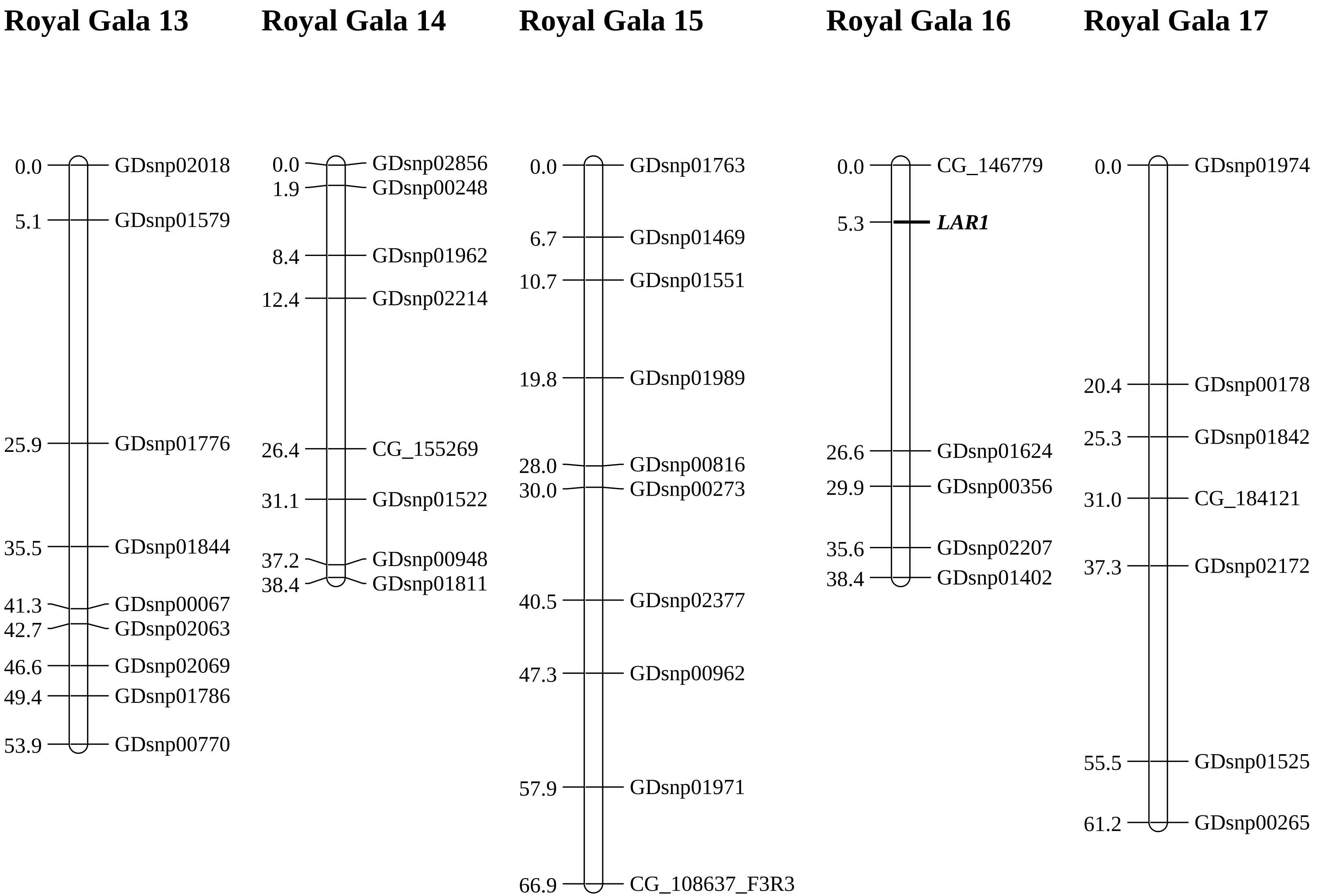
Royal Gala 13
0.0 GDsnp02018
5.1 GDsnp01579
25.9 GDsnp01776
35.5 GDsnp01844
41.3 GDsnp00067
42.7 GDsnp02063
46.6 GDsnp02069
49.4 GDsnp01786
53.9 GDsnp00770
Royal Gala 14
0.0 GDsnp02856
1.9 GDsnp00248
8.4 GDsnp01962
12.4 GDsnp02214
26.4 CG_155269
31.1 GDsnp01522
37.2 GDsnp00948
38.4 GDsnp01811
Royal Gala 15
0.0 GDsnp01763
6.7 GDsnp01469
10.7 GDsnp01551
19.8 GDsnp01989
28.0 GDsnp00816
30.0 GDsnp00273
40.5 GDsnp02377
47.3 GDsnp00962
57.9 GDsnp01971
66.9 CG_108637_F3R3
Royal Gala 16
0.0 CG_146779
5.3 LAR1
26.6 GDsnp01624
29.9 GDsnp00356
35.6 GDsnp02207
38.4 GDsnp01402
Royal Gala 17
0.0 GDsnp01974
20.4 GDsnp00178
25.3 GDsnp01842
31.0 CG_184121
37.3 GDsnp02172
55.5 GDsnp01525
61.2 GDsnp00265

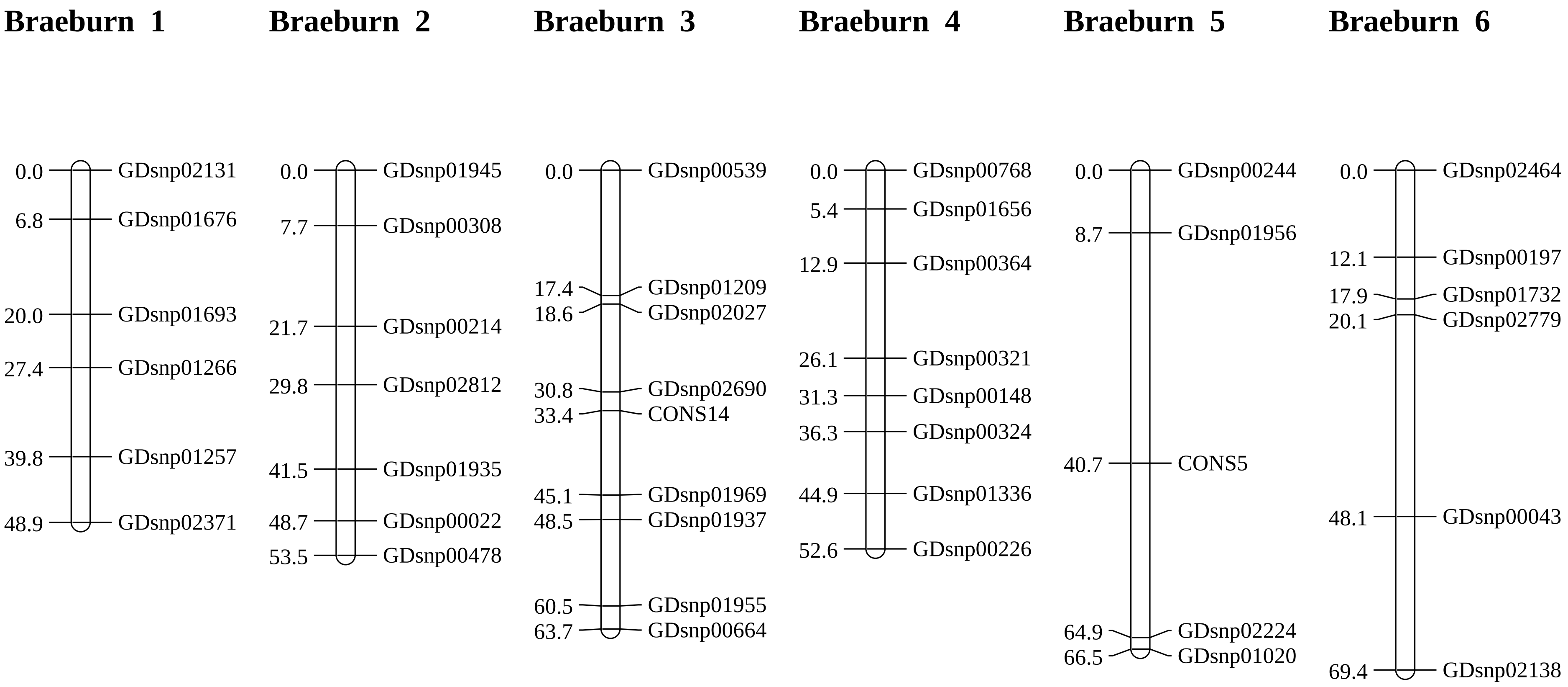
Braeburn 1
0.0 GDsnp02131
6.8 GDsnp01676
20.0 GDsnp01693
27.4 GDsnp01266
39.8 GDsnp01257
48.9 GDsnp02371
Braeburn 2
0.0 GDsnp01945
7.7 GDsnp00308
21.7 GDsnp00214
29.8 GDsnp02812
41.5 GDsnp01935
48.7 GDsnp00022
53.5 GDsnp00478
Braeburn 3
0.0 GDsnp00539
17.4 GDsnp01209
18.6 GDsnp02027
30.8 GDsnp02690
33.4 CONS14
45.1 GDsnp01969
48.5 GDsnp01937
60.5 GDsnp01955
63.7 GDsnp00664
Braeburn 4
0.0 GDsnp00768
5.4 GDsnp01656
12.9 GDsnp00364
26.1 GDsnp00321
31.3 GDsnp00148
36.3 GDsnp00324
44.9 GDsnp01336
52.6 GDsnp00226
Braeburn 5
0.0 GDsnp00244
8.7 GDsnp01956
40.7 CONS5
64.9 GDsnp02224
66.5 GDsnp01020
Braeburn 6
0.0 GDsnp02464
12.1 GDsnp00197
17.9 GDsnp01732
20.1 GDsnp02779
48.1 GDsnp00043
69.4 GDsnp02138

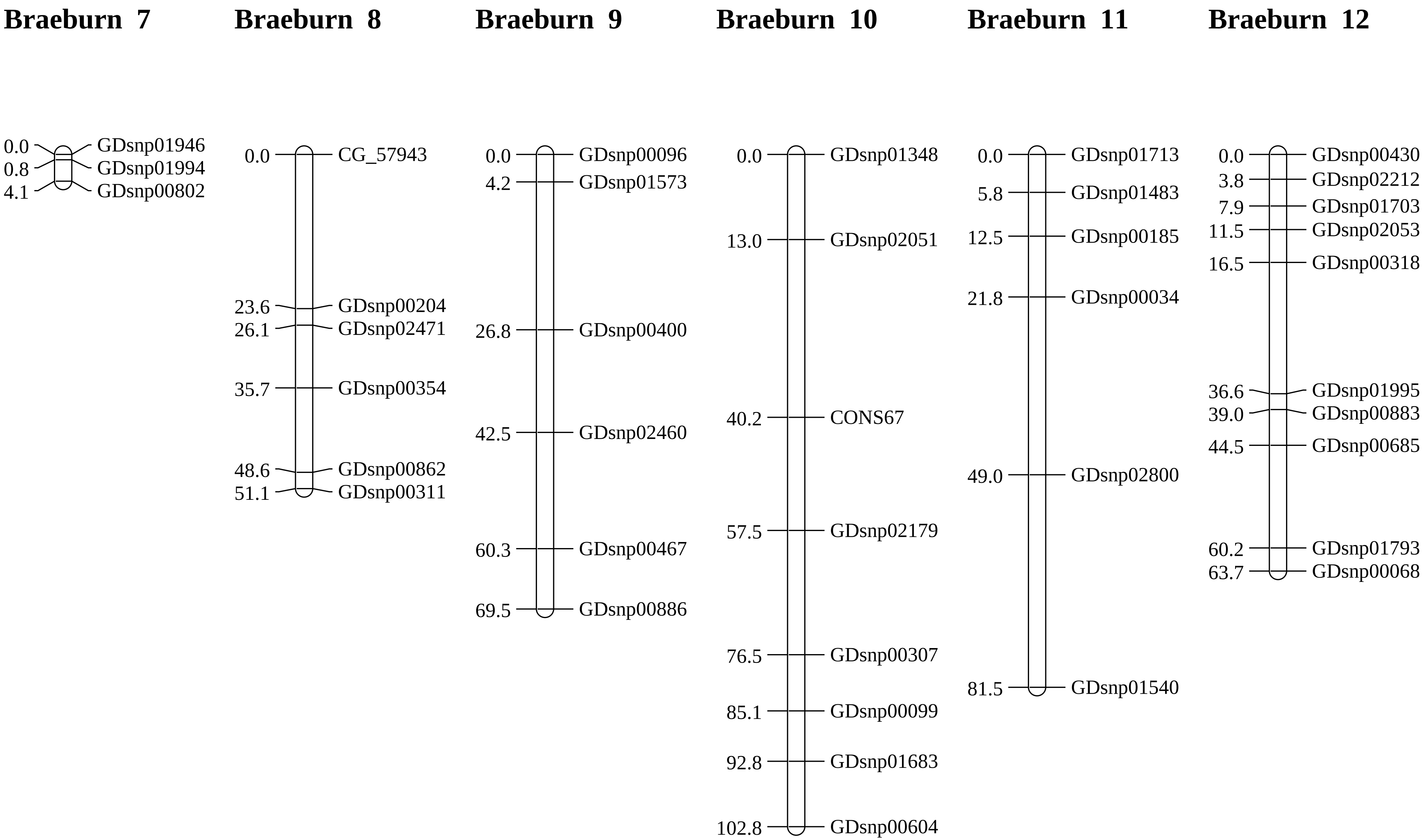

Braeburn 7
0.0 GDsnp01946
0.8 GDsnp01994
4.1 GDsnp00802
Braeburn 8
0.0 CG_57943
23.6 GDsnp00204
26.1 GDsnp02471
35.7 GDsnp00354
48.6 GDsnp00862
51.1 GDsnp00311
Braeburn 9
0.0 GDsnp00096
4.2 GDsnp01573
26.8 GDsnp00400
42.5 GDsnp02460
60.3 GDsnp00467
69.5 GDsnp00886
Braeburn 10
0.0 GDsnp01348
13.0 GDsnp02051
40.2 CONS67
57.5 GDsnp02179
76.5 GDsnp00307
85.1 GDsnp00099
92.8 GDsnp01683
102.8 GDsnp00604
Braeburn 11
0.0 GDsnp01713
5.8 GDsnp01483
12.5 GDsnp00185
21.8 GDsnp00034
49.0 GDsnp02800
81.5 GDsnp01540
Braeburn 12
0.0 GDsnp00430
3.8 GDsnp02212
7.9 GDsnp01703
11.5 GDsnp02053
16.5 GDsnp00318
36.6 GDsnp01995
39.0 GDsnp00883
44.5 GDsnp00685
60.2 GDsnp01793
63.7 GDsnp00068

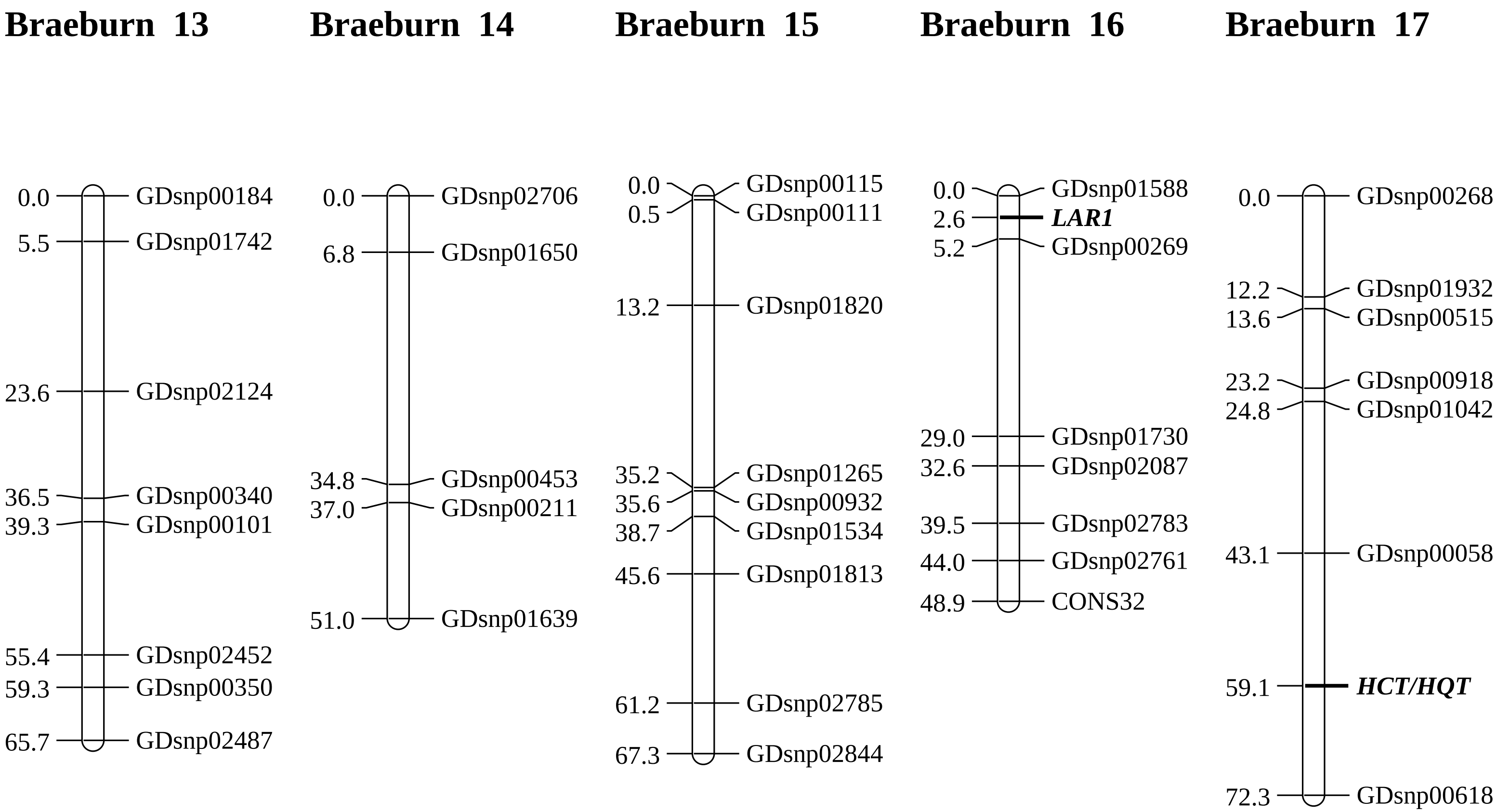
Braeburn 13
0.0 GDsnp00184
5.5 GDsnp01742
23.6 GDsnp02124
36.5 GDsnp00340
39.3 GDsnp00101
55.4 GDsnp02452
59.3 GDsnp00350
65.7 GDsnp02487
Braeburn 14
0.0 GDsnp02706
6.8 GDsnp01650
34.8 GDsnp00453
37.0 GDsnp00211
51.0 GDsnp01639
Braeburn 15
0.0 GDsnp00115
0.5 GDsnp00111
13.2 GDsnp01820
35.2 GDsnp01265
35.6 GDsnp00932
38.7 GDsnp01534
45.6 GDsnp01813
61.2 GDsnp02785
67.3 GDsnp02844
Braeburn 16
0.0 GDsnp01588
2.6 LAR1
5.2 GDsnp00269
29.0 GDsnp01730
32.6 GDsnp02087
39.5 GDsnp02783
44.0 GDsnp02761
48.9 CONS32
Braeburn 17
0.0 GDsnp00268
12.2 GDsnp01932
13.6 GDsnp00515
23.2 GDsnp00918
24.8 GDsnp01042
43.1 GDsnp00058
59.1 HCT/HQT
72.3 GDsnp00618
