# Supplementary material for: QTL and candidate gene mapping for polyphenolic composition in apple fruit
Source: BMC Plant Biol. 2012 Jan 23;12:12. doi: 10.1186/1471-2229-12-12 (PMC3285079; doi:10.1186/1471-2229-12-12)
Supplement: Additional File 4 — figure S3: A simplified schematic of the polyphenolics synthesis pathway. Gene symbols are identified in Table 3. [file 1471-2229-12-12-S4.PDF]

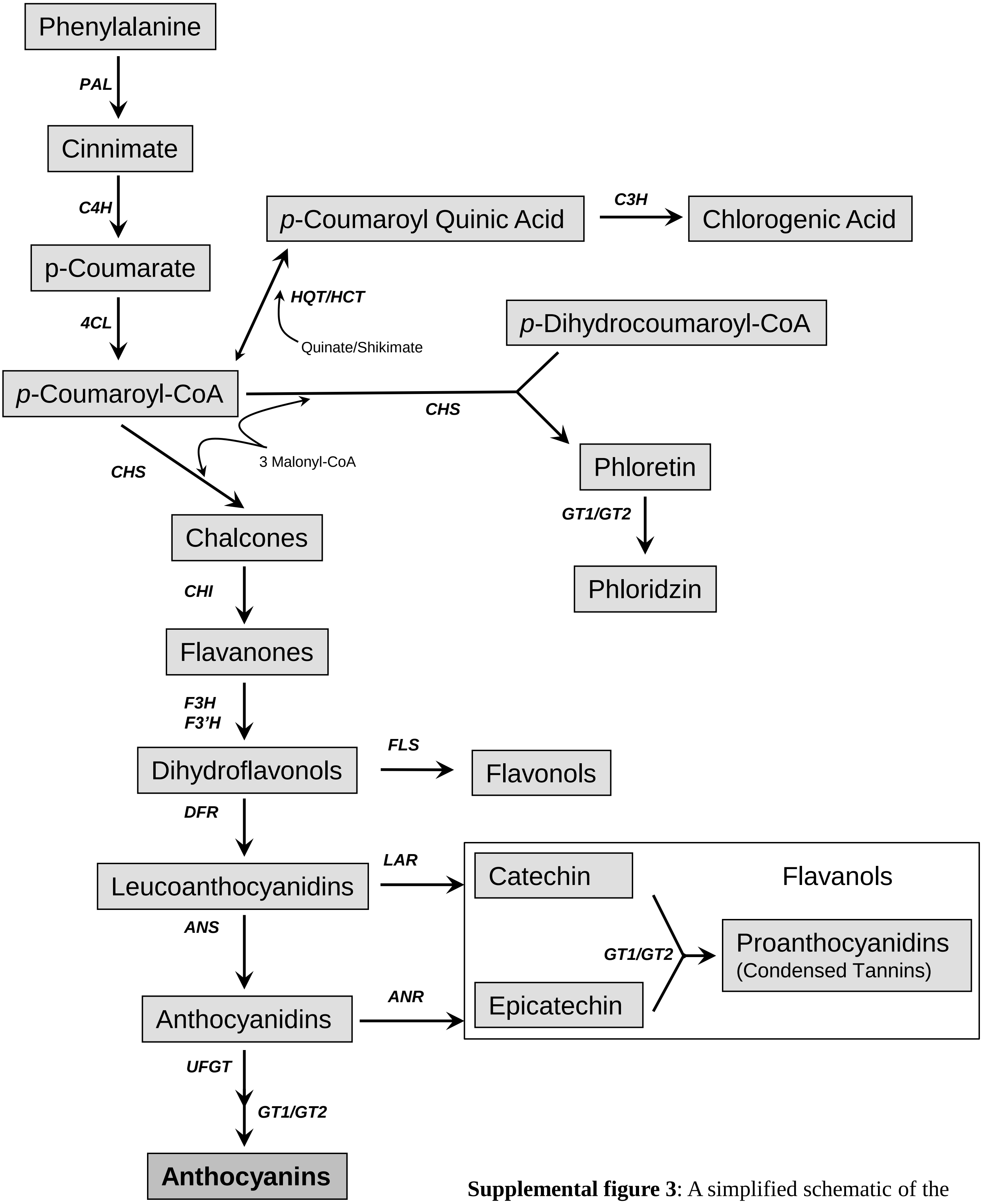

Supplemental figure 3: A simplified schematic of the polyphenolics synthesis pathway. Gene symbols are identified in Table 3.
