# Supplementary material for: QTL and candidate gene mapping for polyphenolic composition in apple fruit
Source: BMC Plant Biol. 2012 Jan 23;12:12. doi: 10.1186/1471-2229-12-12 (PMC3285079; doi:10.1186/1471-2229-12-12)
Supplement: Additional File 5 — figure S4: Apple gene predictions found in the QTL interval for flavanol concentration. The figure is depicted as a set of GBrowser tracks showing the position on LG 16 (top track), close up position (second and third tracks), apple genome assembly contigs (fourth track), IASMA predicted apple gene set and apple cDNA sequences (fifth track; Malus Bioview™) [37] mapping to the region (positioned using GMAP). Gene names are indicated where known. [file 1471-2229-12-12-S5.PDF]

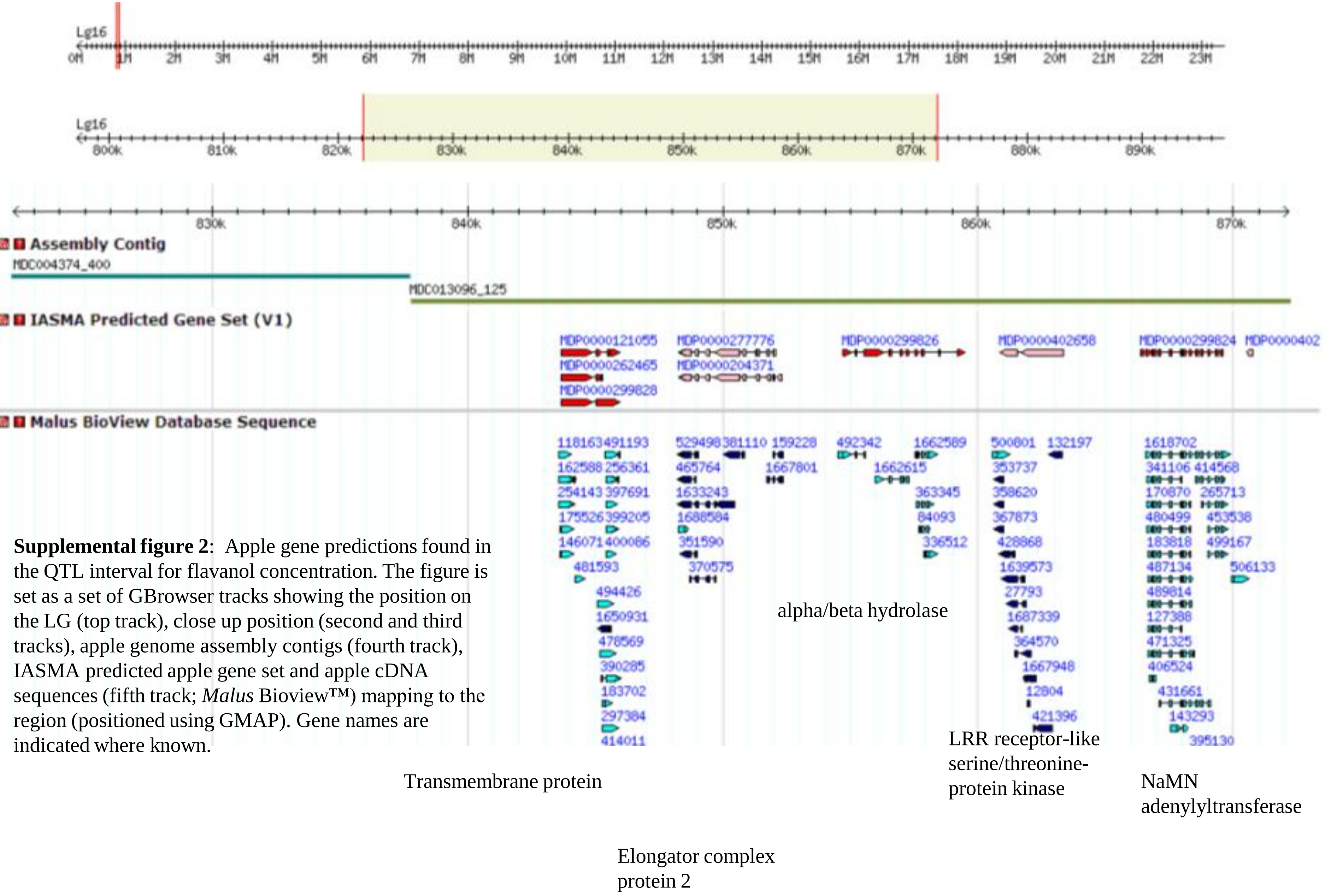

Supplemental figure 2: Apple gene predictions found in the QTL interval for flavanol concentration. The figure is set as a set of GBrowser tracks showing the position on the LG (top track), close up position (second and third tracks), apple genome assembly contigs (fourth track), IASMA predicted apple gene set and apple cDNA sequences (fifth track; *Malus* Bioview™) mapping to the region (positioned using GMAP). Gene names are indicated where known.

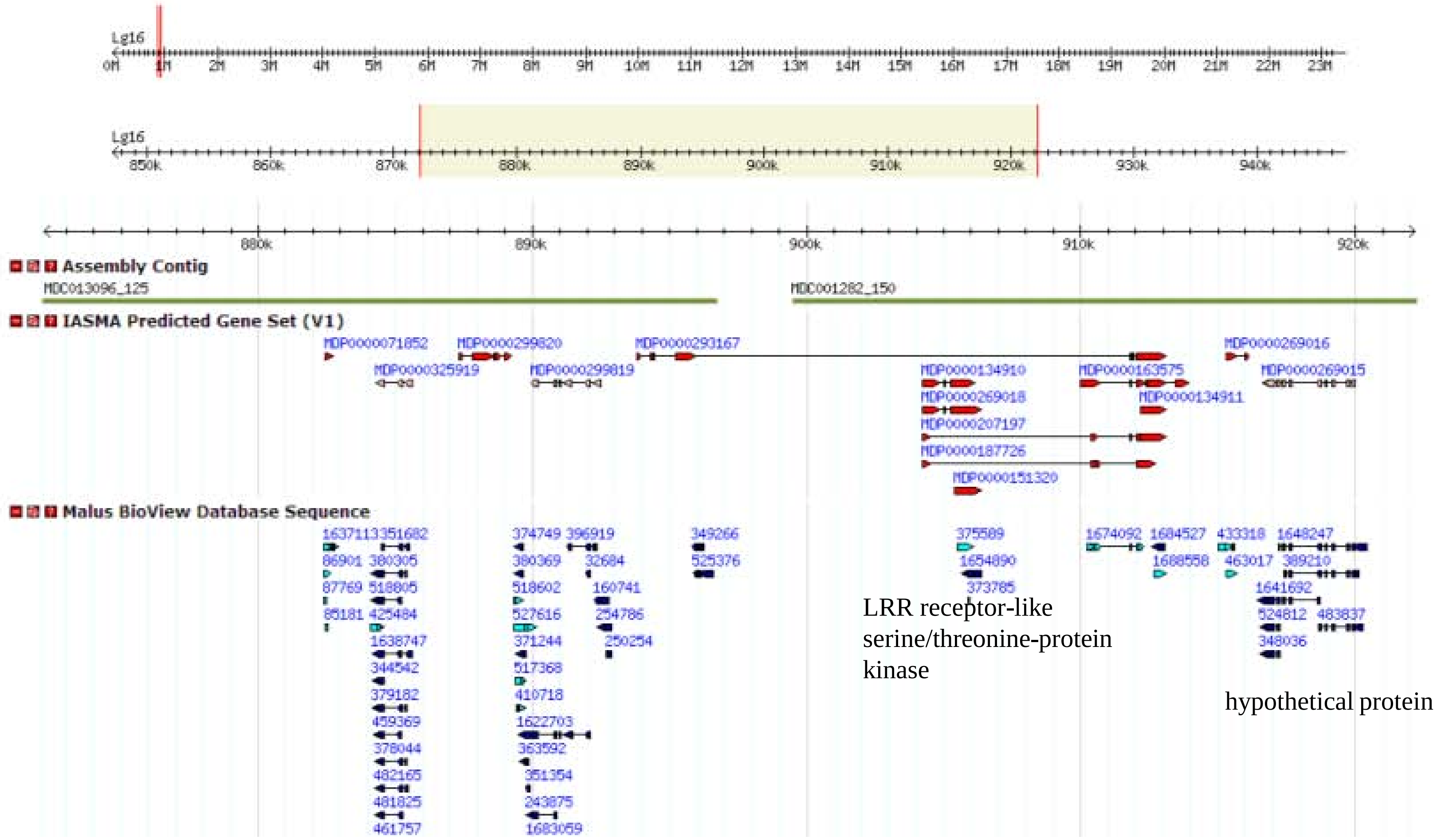

LRR receptor-like serine/threonine-protein kinase

hypothetical protein

Protein lyl-1

lipid-transfer protein
AKCS9

hypothetical protein

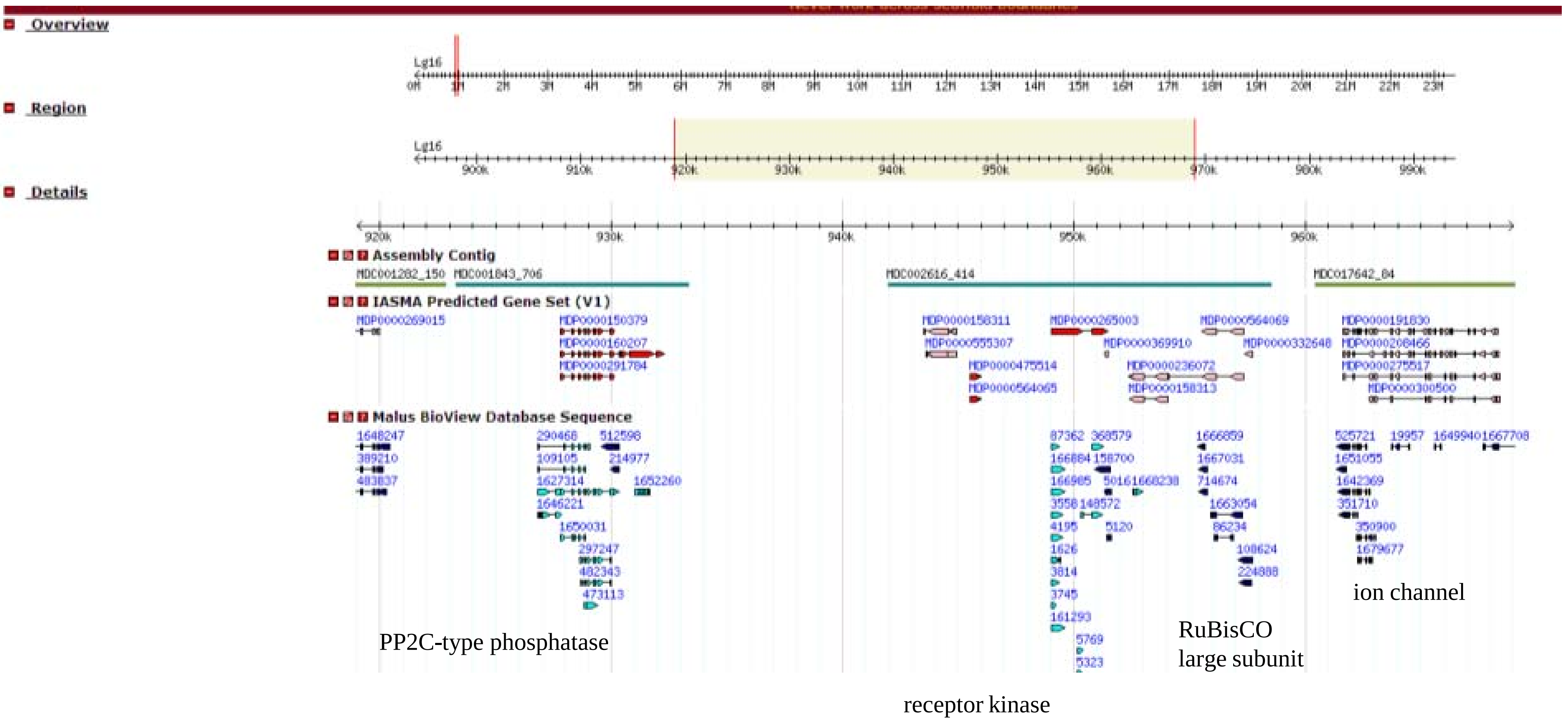
Overview
Region
Details
Assembly Contig
IASMA Predicted Gene Set (V1)
Malus BioView Database Sequence
PP2C-type phosphatase
receptor kinase
RuBisCO
large subunit
ion channel

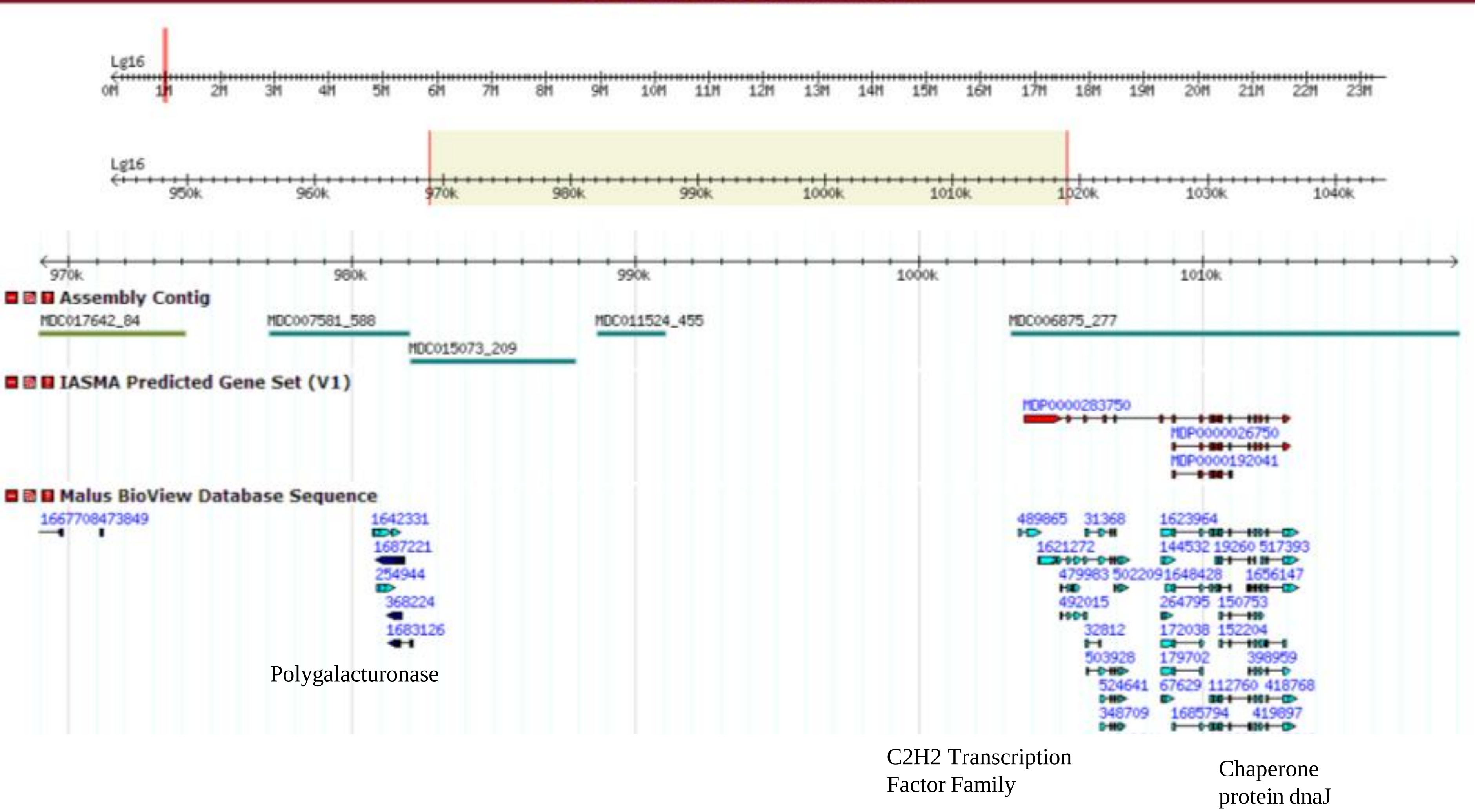
Lg16
Assembly Contig
MDC017642_84
MDC007581_588
MDC015073_209
MDC011524_455
MDC006875_277
IASMA Predicted Gene Set (V1)
MDP0000283750
MDP0000026750
MDP0000192041
Malus BioView Database Sequence
Polygalacturonase
C2H2 Transcription Factor Family
Chaperone protein dnaJ

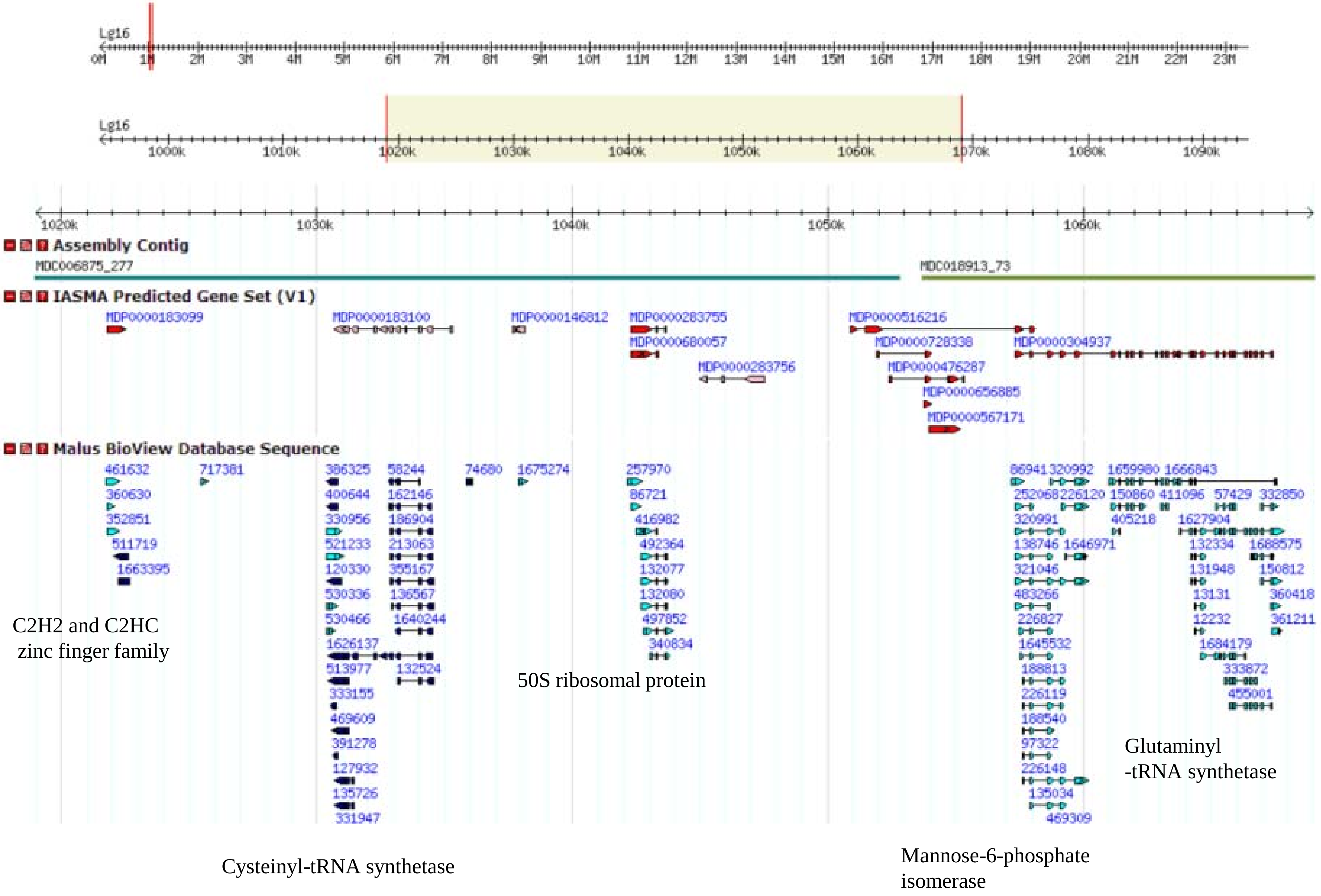
Lg16
0M 1M 2M 3M 4M 5M 6M 7M 8M 9M 10M 11M 12M 13M 14M 15M 16M 17M 18M 19M 20M 21M 22M 23M
Lg16
1000k 1010k 1020k 1030k 1040k 1050k 1060k 1070k 1080k 1090k
1020k 1030k 1040k 1050k 1060k
Assembly Contig
MDC006875_277
MDC018913_73
IASMA Predicted Gene Set (V1)
MDP0000183099
MDP0000183100
MDP0000146812
MDP0000283755
MDP0000680057
MDP0000283756
MDP0000516216
MDP0000728338
MDP0000304937
MDP0000476287
MDP0000656885
MDP0000567171
Malus BioView Database Sequence
461632 360630 352851 511719 1663395
717381
386325 400644 330956 521233 120330 530336 530466 1626137 513977 333155 469609 391278 127932 135726 331947
58244 162146 186904 213063 355167 136567 1640244 132524
74680
1675274
257970 86721 416982 492364 132077 132080 497852 340834
86941 320992 252068 226120 320991 138746 1646971 321046 483266 226827 1645532 188813 226119 188540 97322 226148 135034 469309
1659980 1666843 150860 411096 57429 332850 405218 1627904 132334 1688575 131948 150812 13131 360418 12232 361211 1684179 333872 455001
C2H2 and C2HC zinc finger family
50S ribosomal protein
Glutaminyl -tRNA synthetase
Cysteinyl-tRNA synthetase
Mannose-6-phosphate isomerase

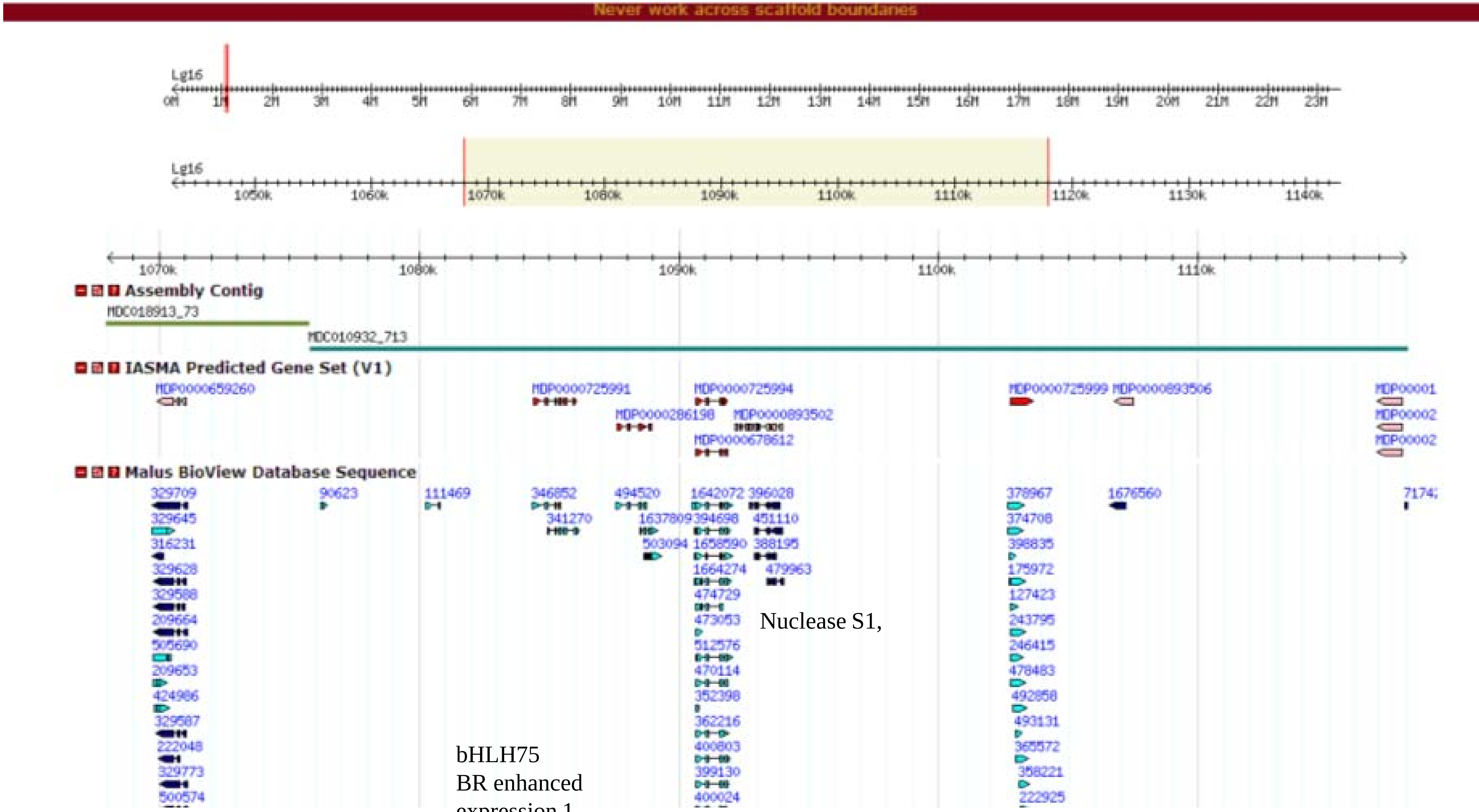

Nuclease S1,

bHLH75
BR enhanced
expression 1

MdMYB7

stress protein

hsl protease ATP-binding

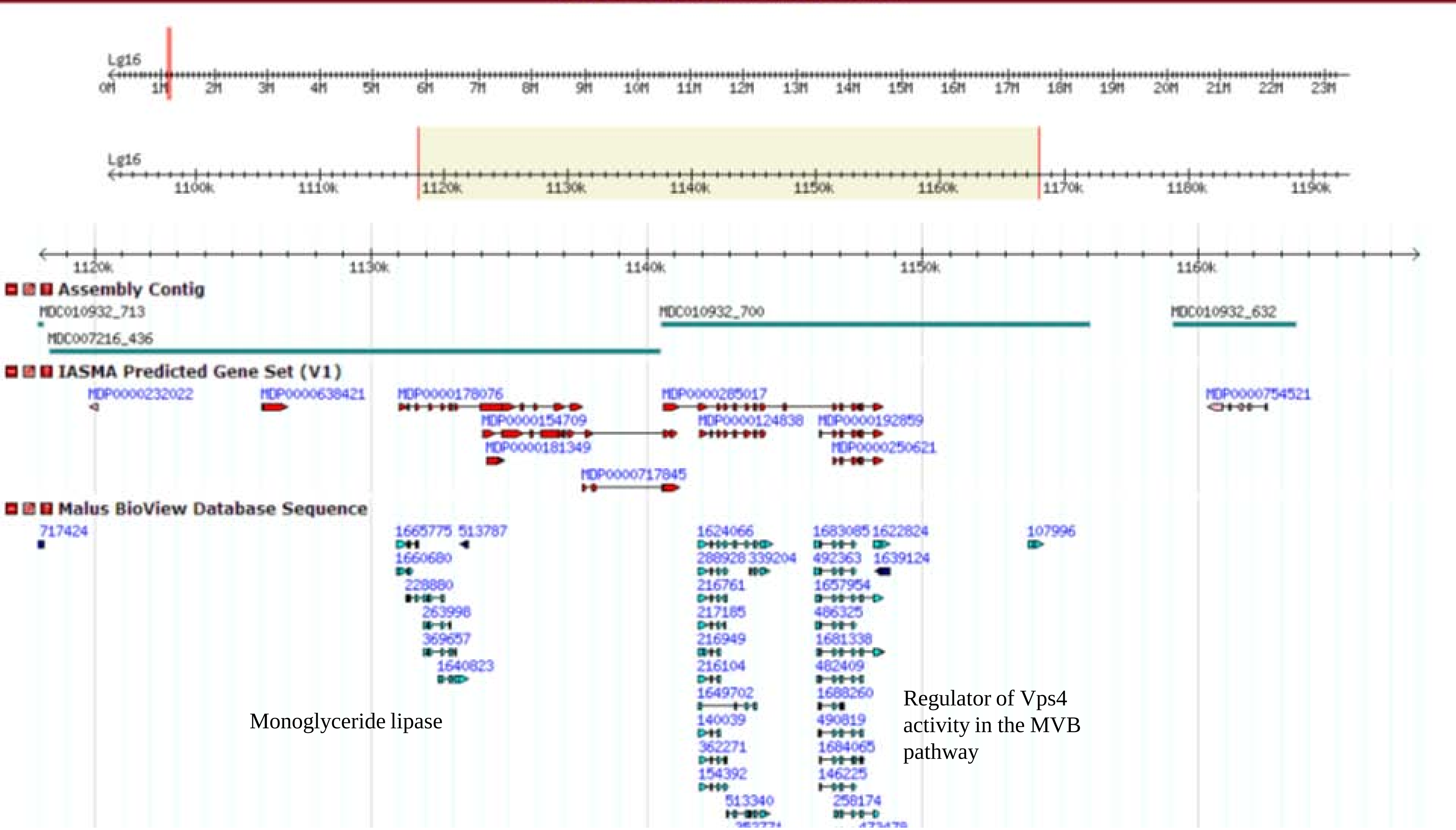

Monoglyceride lipase (MGL)

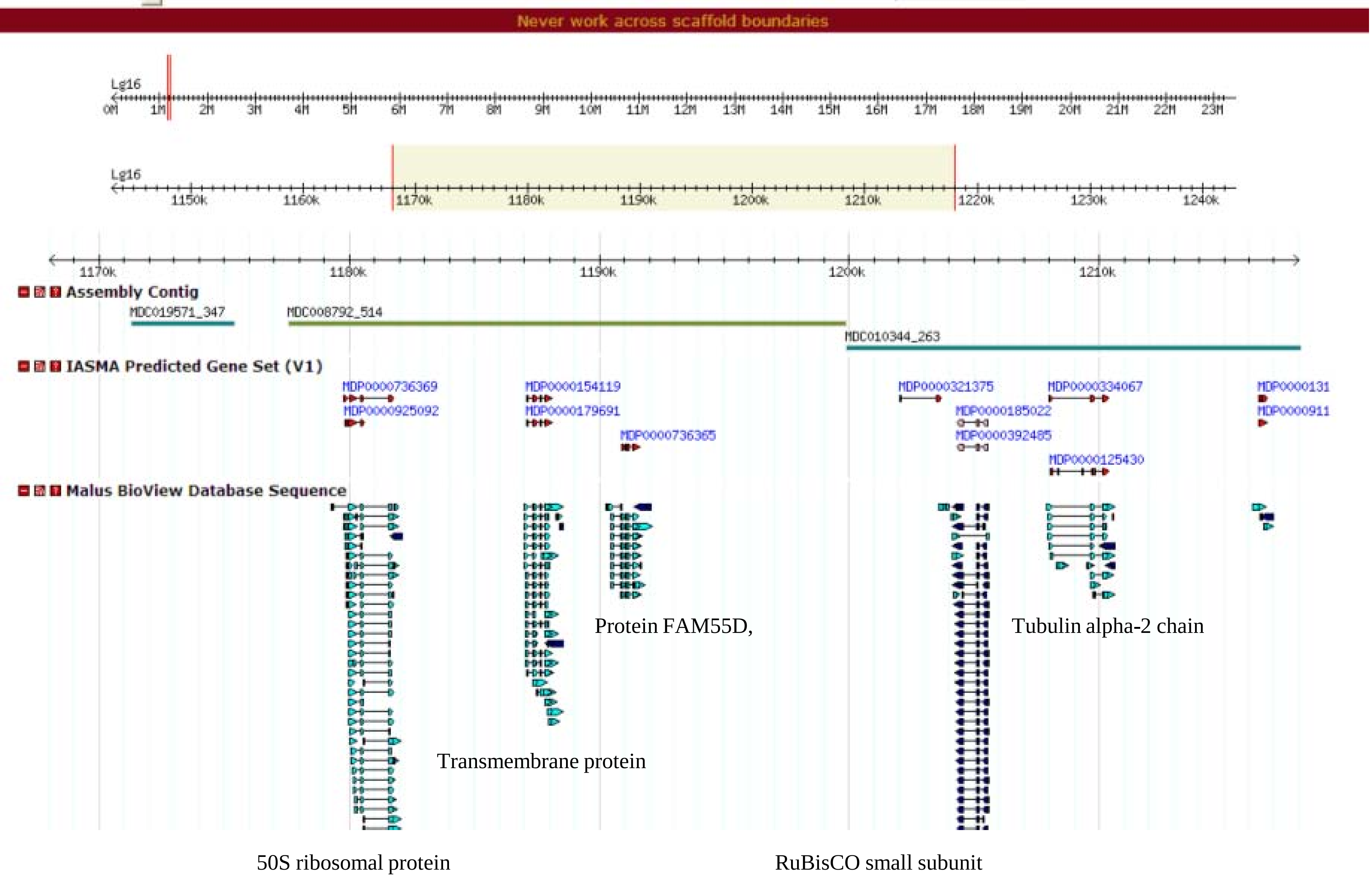
Never work across scaffold boundaries
Lg16
Assembly Contig
MDC019571_347
MDC008792_514
MDC010344_263
IASMA Predicted Gene Set (V1)
MDP0000736369
MDP0000925092
MDP0000154119
MDP0000179691
MDP0000736365
MDP0000321375
MDP0000185022
MDP0000392485
MDP0000334067
MDP0000125430
MDP0000131
MDP0000911
Malus BioView Database Sequence
Protein FAM55D,
Tubulin alpha-2 chain
Transmembrane protein
50S ribosomal protein
RuBisCO small subunit

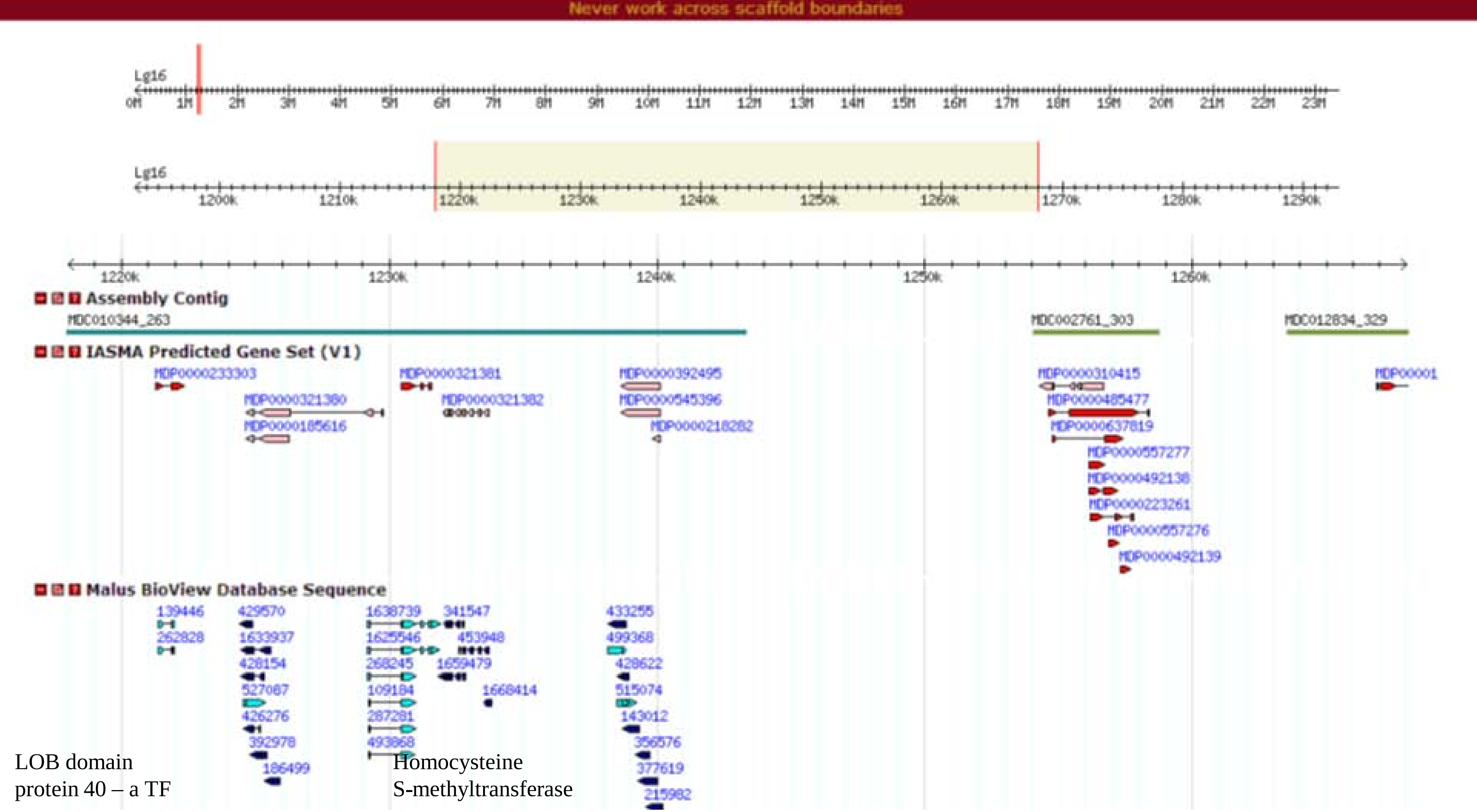

LOB domain
protein 40 – a TF

Homocysteine
S-methyltransferase

3-ketoacyl-CoA
synthase 6 (KCS-6)

**Zinc finger protein
CONSTANS-LIKE 6**

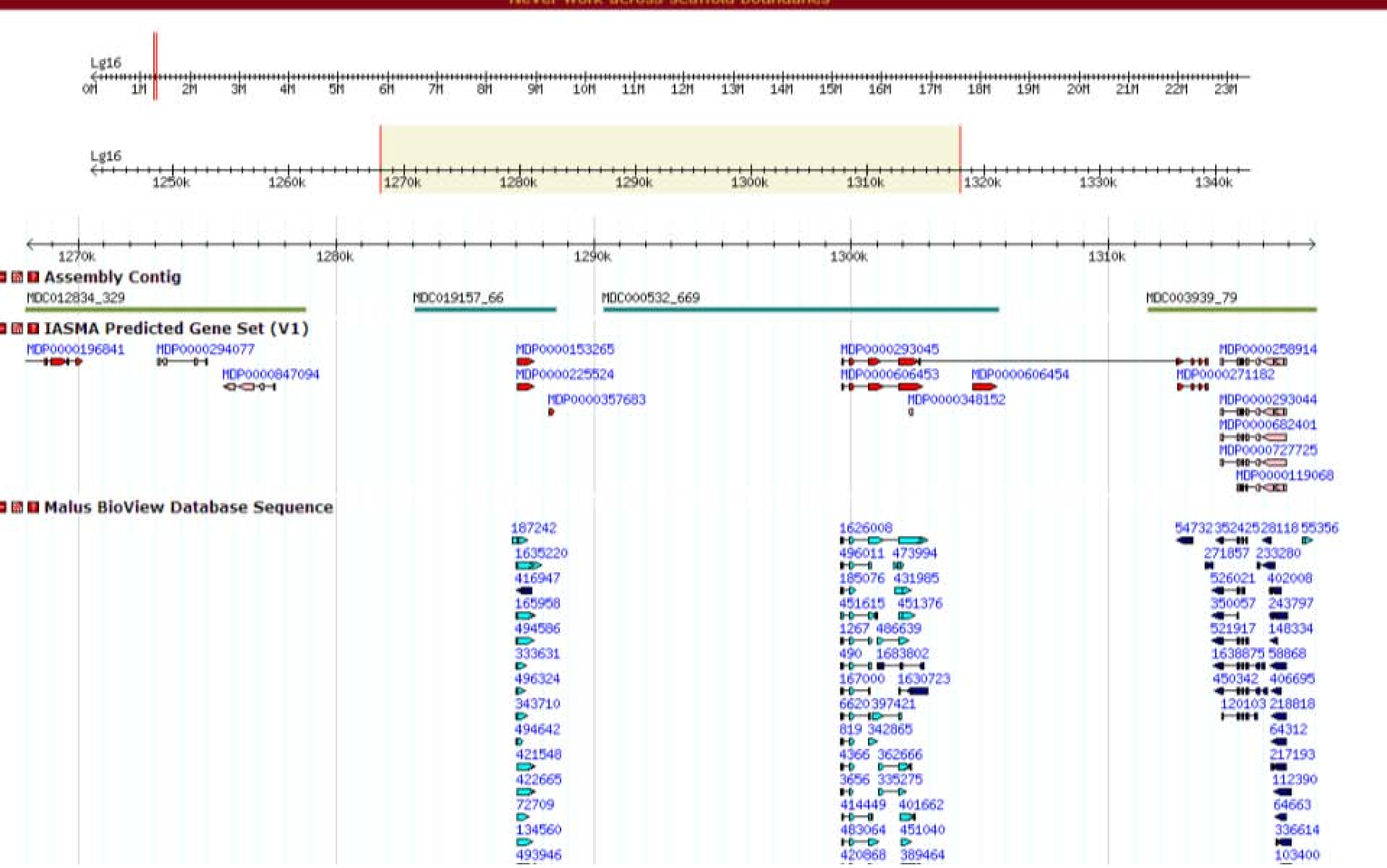

Glutathione S-transferase 6

Peptide transporter PTR1

Dihydrolipoyllysine-residue acetyltransferase

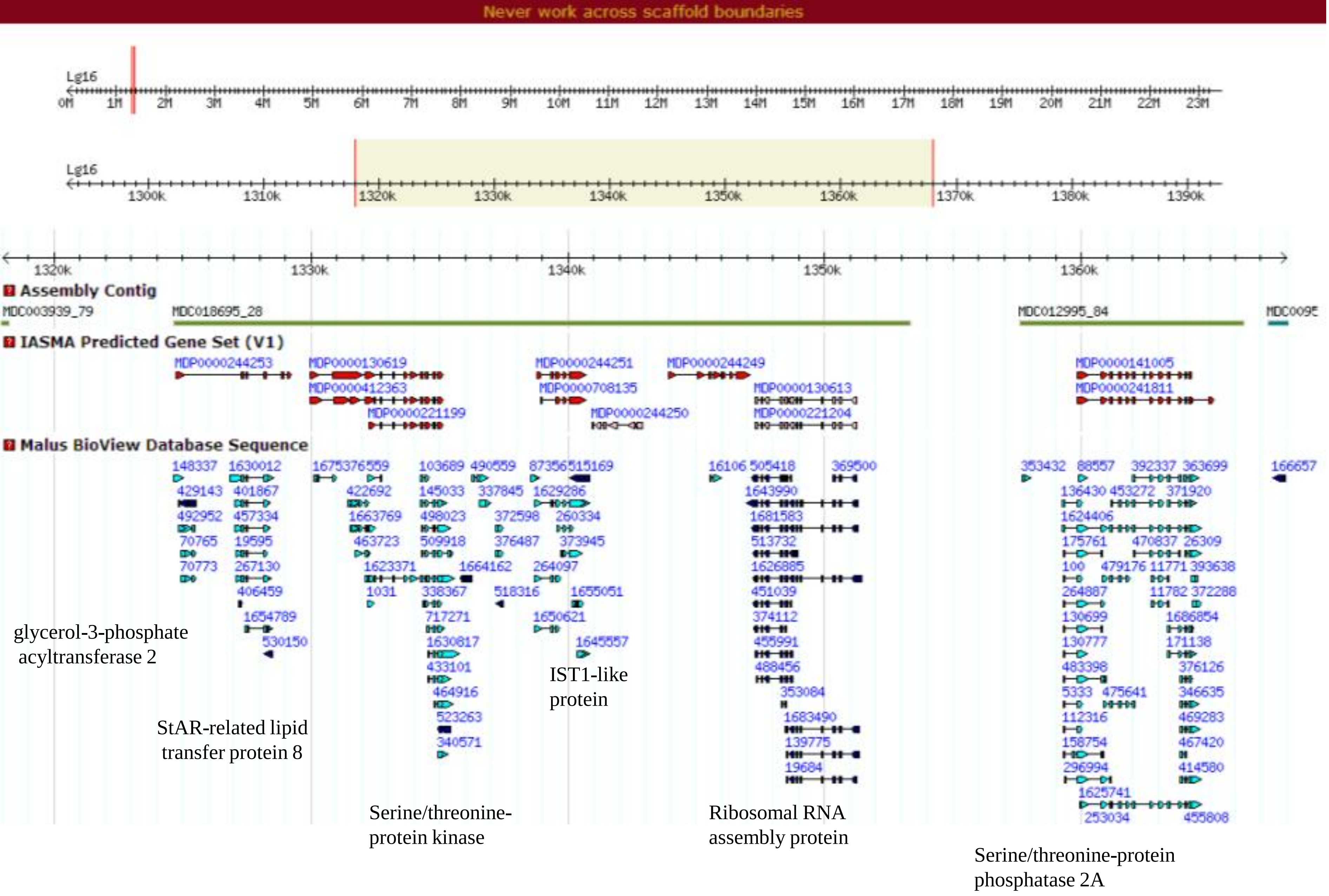
Never work across scaffold boundaries
Lg16
Lg16
Assembly Contig
MDC003939_79
MDC018695_28
MDC012995_84
IASMA Predicted Gene Set (V1)
MDP0000244253
MDP0000130619
MDP0000412363
MDP0000221199
MDP0000244251
MDP0000708135
MDP0000244250
MDP0000244249
MDP0000130613
MDP0000221204
MDP0000141005
MDP0000241811
Malus BioView Database Sequence
glycerol-3-phosphate acyltransferase 2
StAR-related lipid transfer protein 8
Serine/threonine-protein kinase
IST1-like protein
Ribosomal RNA assembly protein
Serine/threonine-protein phosphatase 2A

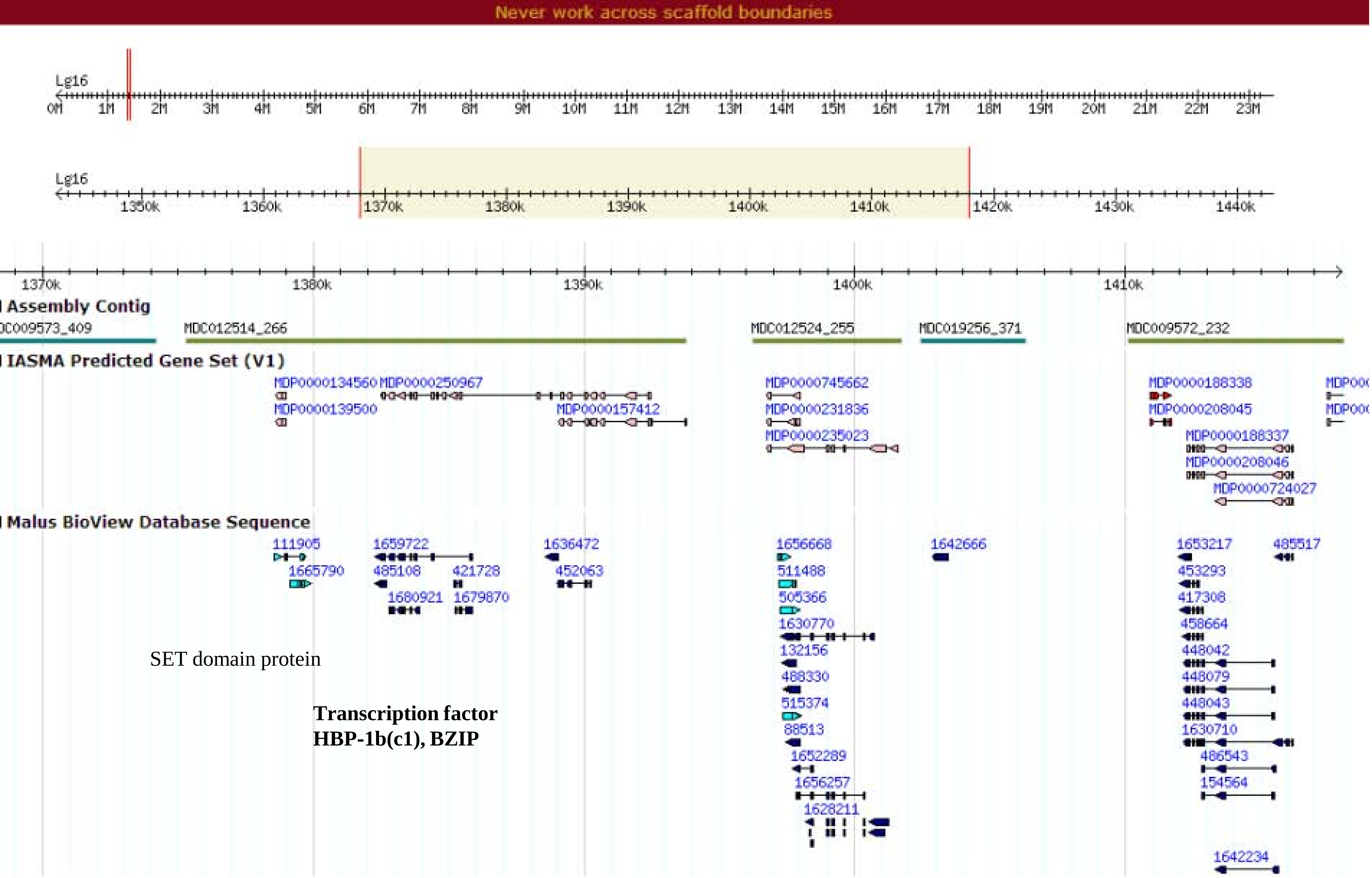

SET domain protein

Transcription factor
HBP-1b(c1), BZIP

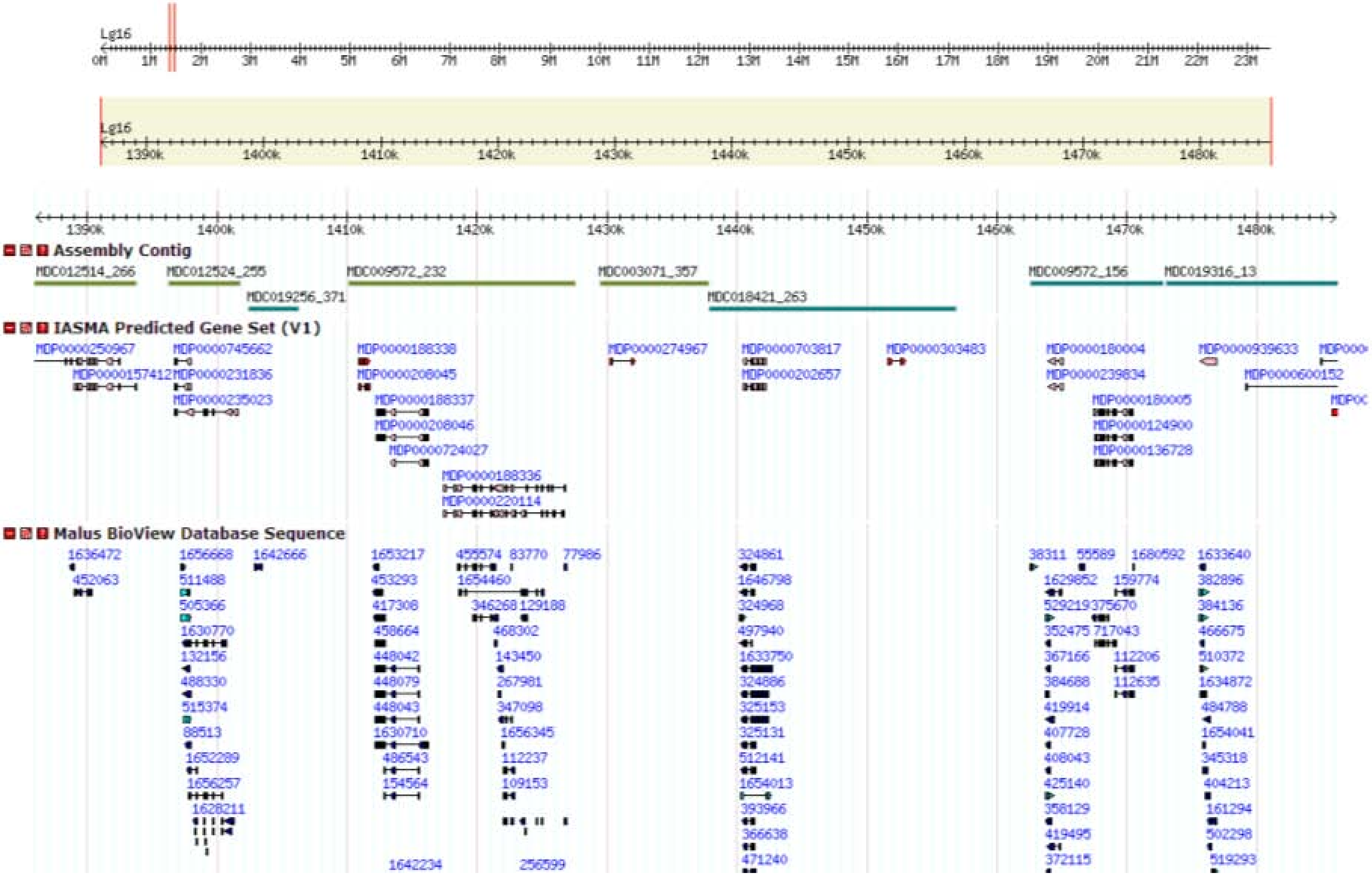

tRNA

Lysine transporter

RNA pol

MdG2L6
ARR18
MYBrelated

Allene oxidase

AP2D36
Regulator of vac ATPase

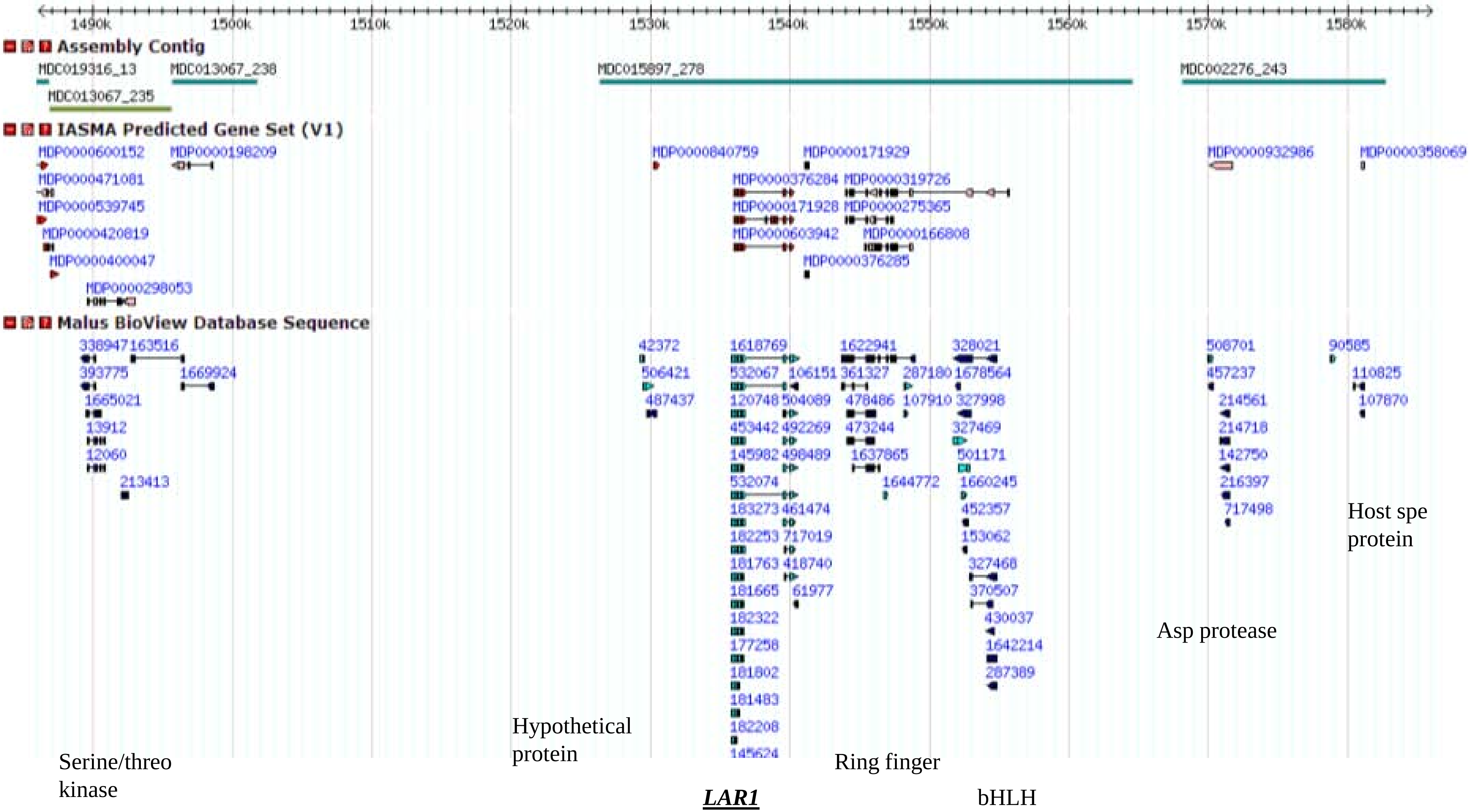

1490k 1500k 1510k 1520k 1530k 1540k 1550k 1560k 1570k 1580k
Assembly Contig
MDC019316_13
MDC013067_238
MDC015897_278
MDC002276_243
MDC013067_235
IASMA Predicted Gene Set (V1)
MDP0000600152
MDP0000198209
MDP0000840759
MDP0000171929
MDP0000932986
MDP0000358069
MDP0000471081
MDP0000376284
MDP0000319726
MDP0000539745
MDP0000171928
MDP0000275365
MDP0000420819
MDP0000603942
MDP0000166808
MDP0000400047
MDP0000376285
MDP0000298053
Malus BioView Database Sequence
Serine/threo kinase
Hypothetical protein
LAR1
Ring finger
bHLH
Asp protease
Host spe protein

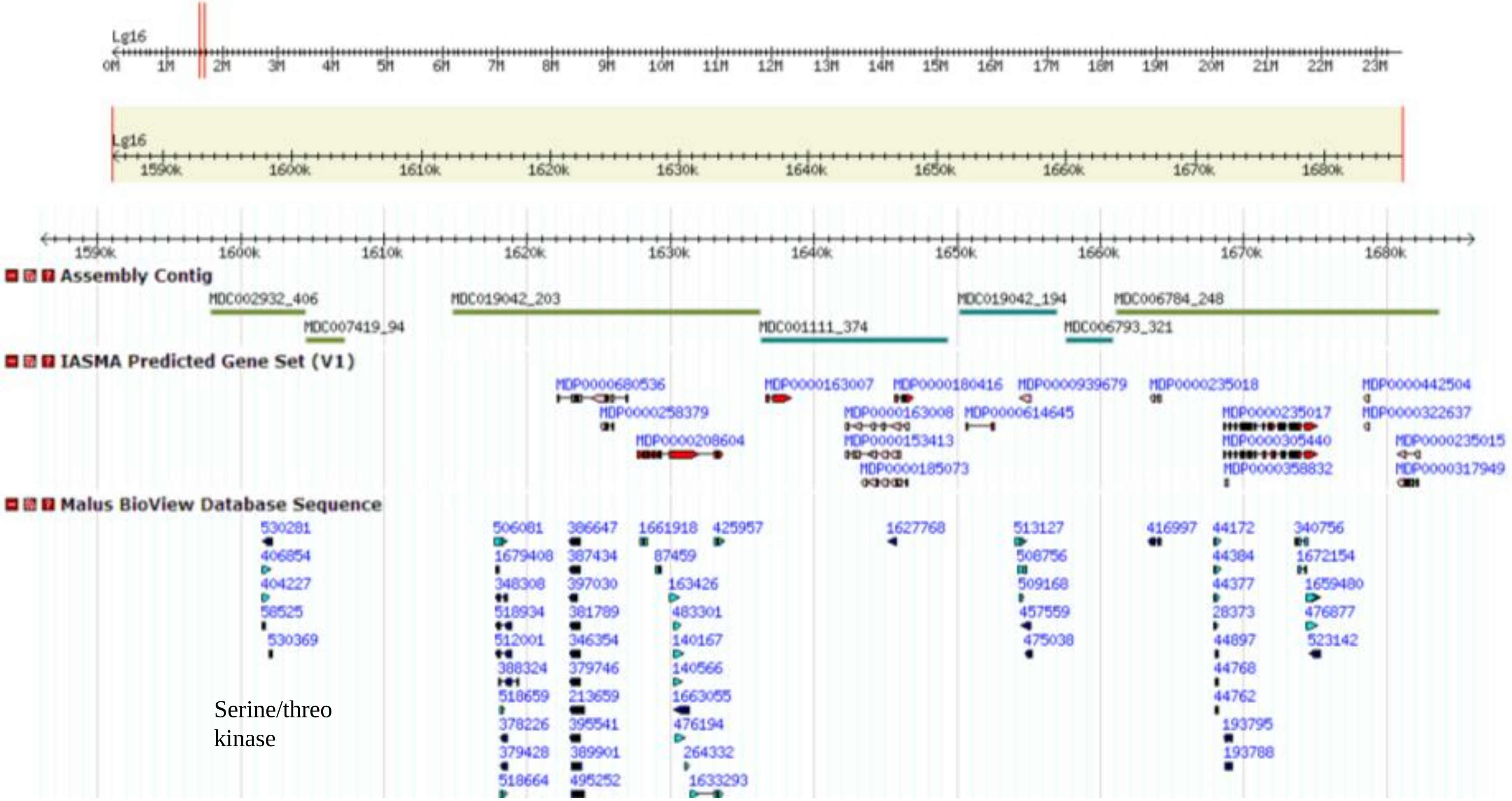

Serine/threo
kinase

rootletin

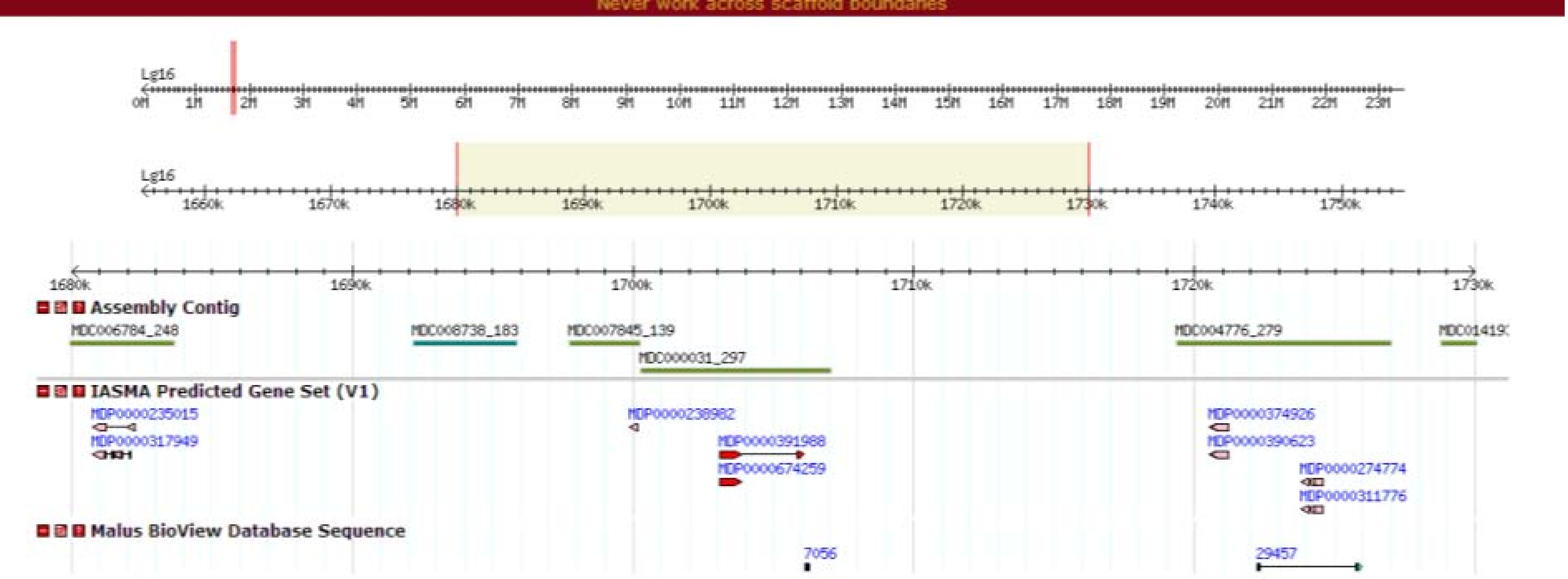

hypothetical protein

Low-density lipoprotein receptor-related protein 5

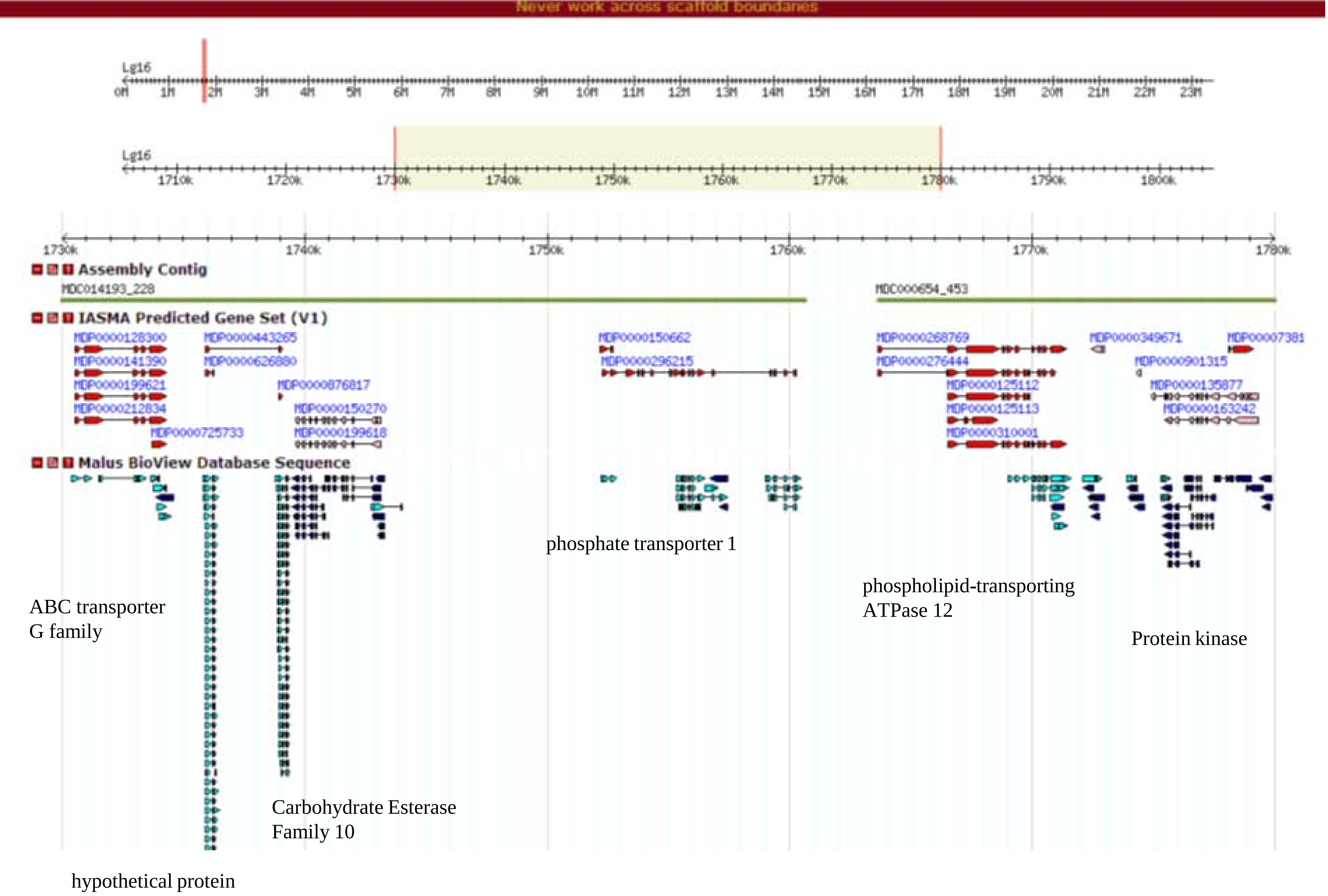

Never work across scaffold boundaries
Lg16
Lg16
Assembly Contig
MDC014193_228
MDC000654_453
IASMA Predicted Gene Set (V1)
MDP0000128300
MDP0000443265
MDP0000150662
MDP0000268769
MDP0000349671
MDP0000141390
MDP0000626880
MDP0000296215
MDP0000276444
MDP0000901315
MDP0000199621
MDP0000876817
MDP0000125112
MDP0000135877
MDP0000212834
MDP0000150270
MDP0000125113
MDP0000163242
MDP0000725733
MDP0000199618
MDP0000310001
Malus BioView Database Sequence
phosphate transporter 1
phospholipid-transporting ATPase 12
ABC transporter G family
Protein kinase
Carbohydrate Esterase Family 10
hypothetical protein

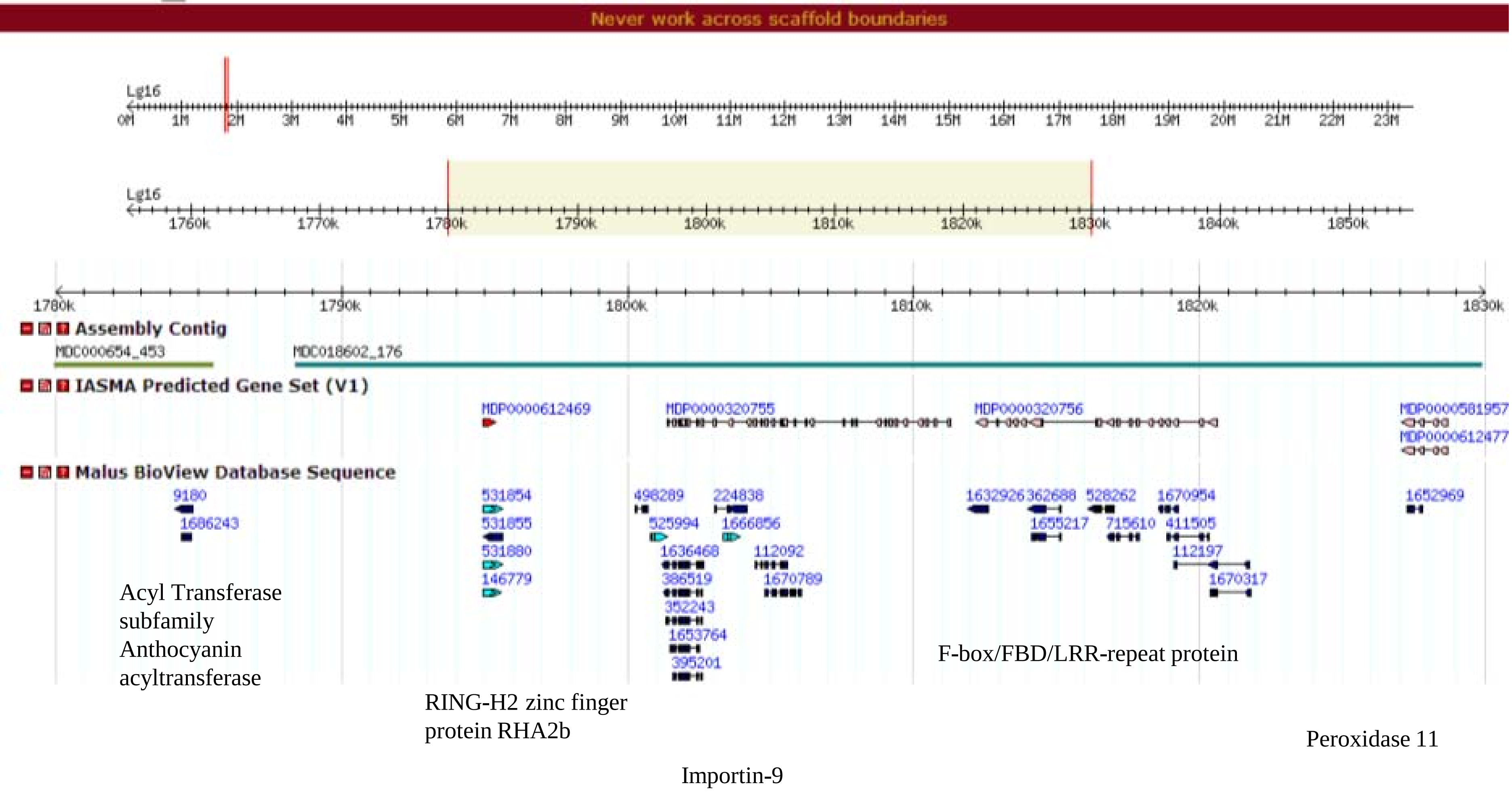
Never work across scaffold boundaries
Lg16
0M 1M 2M 3M 4M 5M 6M 7M 8M 9M 10M 11M 12M 13M 14M 15M 16M 17M 18M 19M 20M 21M 22M 23M
Lg16
1760k 1770k 1780k 1790k 1800k 1810k 1820k 1830k 1840k 1850k
1780k 1790k 1800k 1810k 1820k 1830k
Assembly Contig
MDC000654_453
MDC018602_176
IASMA Predicted Gene Set (V1)
MDP0000612469
MDP0000320755
MDP0000320756
MDP0000581957
MDP0000612477
Malus BioView Database Sequence
9180
1686243
531854
531855
531880
146779
498289
224838
525994
1666856
1636468
112092
386519
1670789
352243
1653764
395201
1632926
362688
528262
1670954
1655217
715610
411505
112197
1670317
1652969
Acyl Transferase subfamily Anthocyanin acyltransferase
RING-H2 zinc finger protein RHA2b
Importin-9
F-box/FBD/LRR-repeat protein
Peroxidase 11

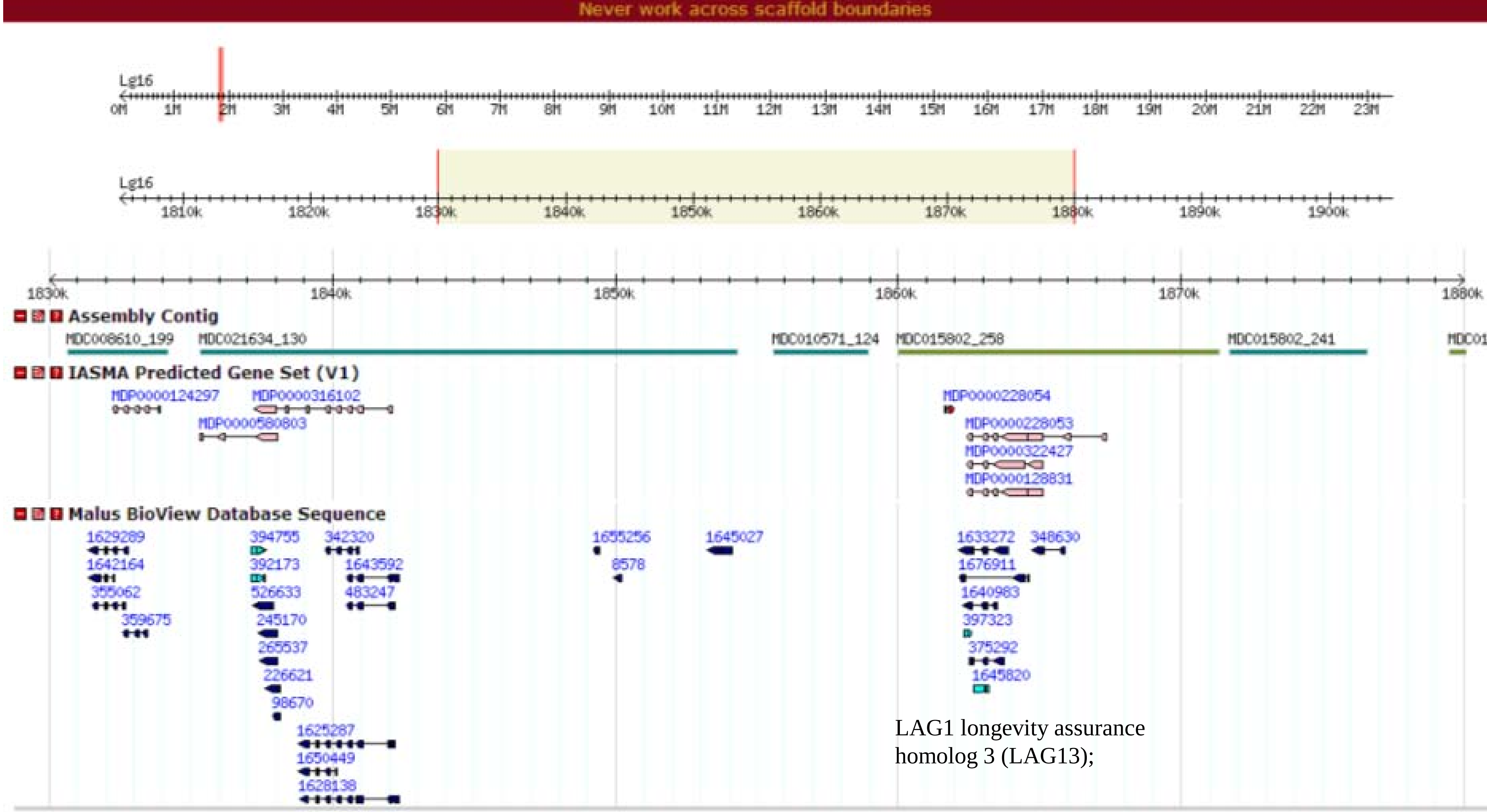

LAG1 longevity assurance homolog 3 (LAG13);

LAG1 longevity assurance homolog 3 (LAG13);

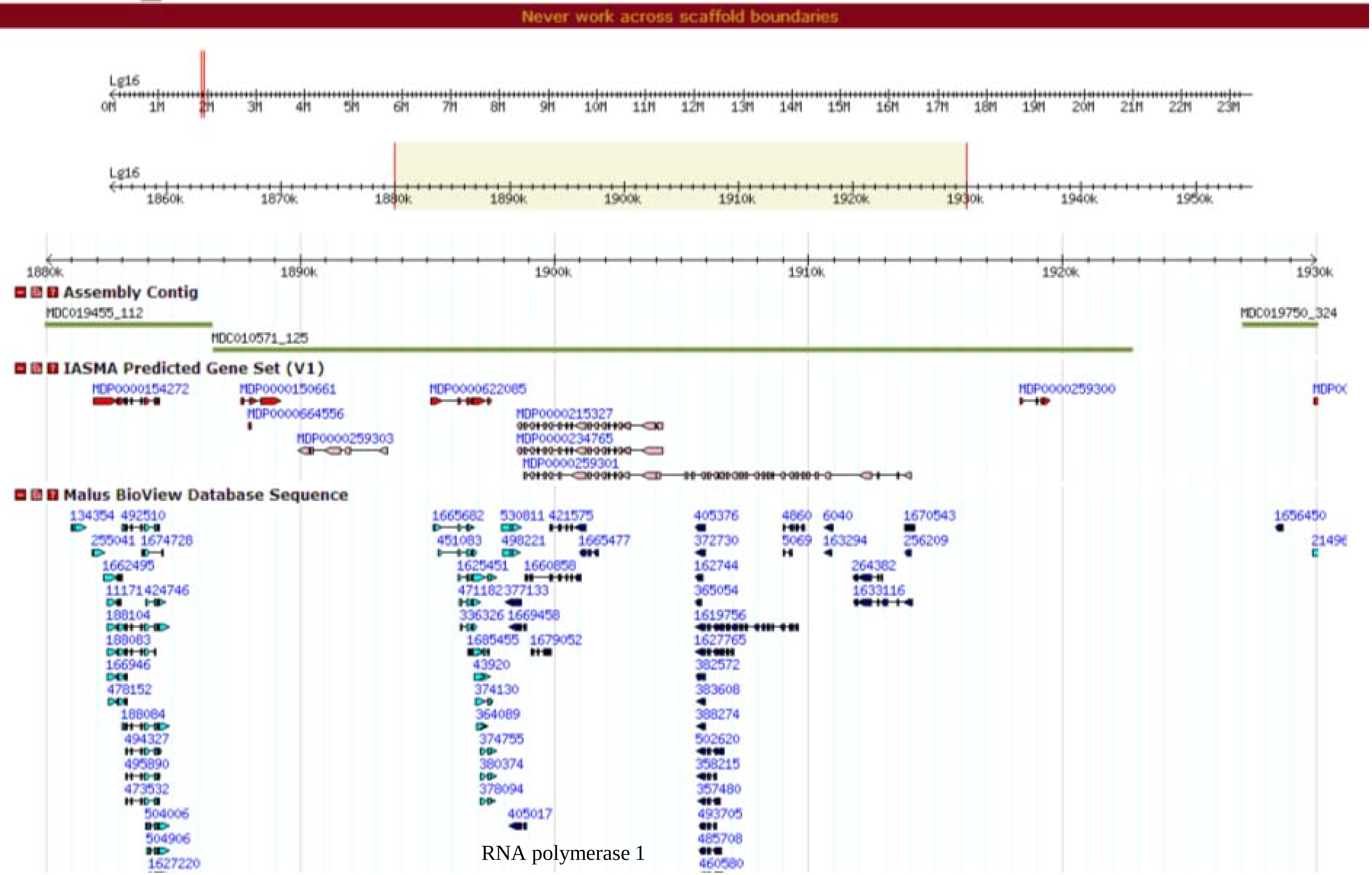

RNA polymerase 1

Transcription factor bHLH49

amino acid transporter 6

Heat shock protein 83

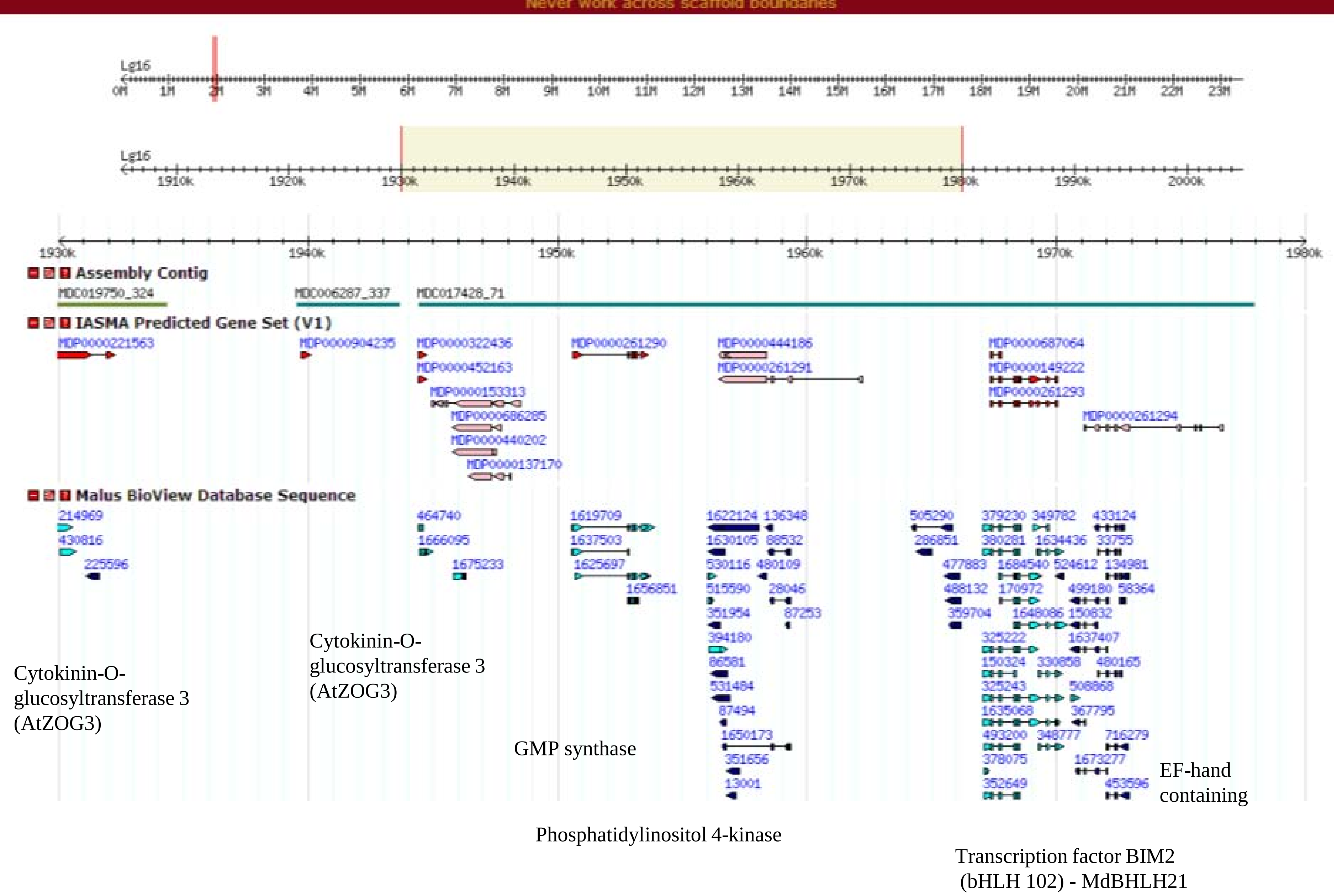
Never work across scaffold boundaries
Lg16
Lg16
Assembly Contig
MDC019750_324
MDC006287_337
MDC017428_71
IASMA Predicted Gene Set (V1)
MDP0000221563
MDP0000904235
MDP0000322436
MDP0000452163
MDP0000153313
MDP0000686285
MDP0000440202
MDP0000137170
MDP0000261290
MDP0000444186
MDP0000261291
MDP0000687064
MDP0000149222
MDP0000261293
MDP0000261294
Malus BioView Database Sequence
Cytokinin-O-glucosyltransferase 3 (AtZOG3)
Cytokinin-O-glucosyltransferase 3 (AtZOG3)
GMP synthase
EF-hand containing
Phosphatidylinositol 4-kinase
Transcription factor BIM2 (bHLH 102) - MdBHLH21

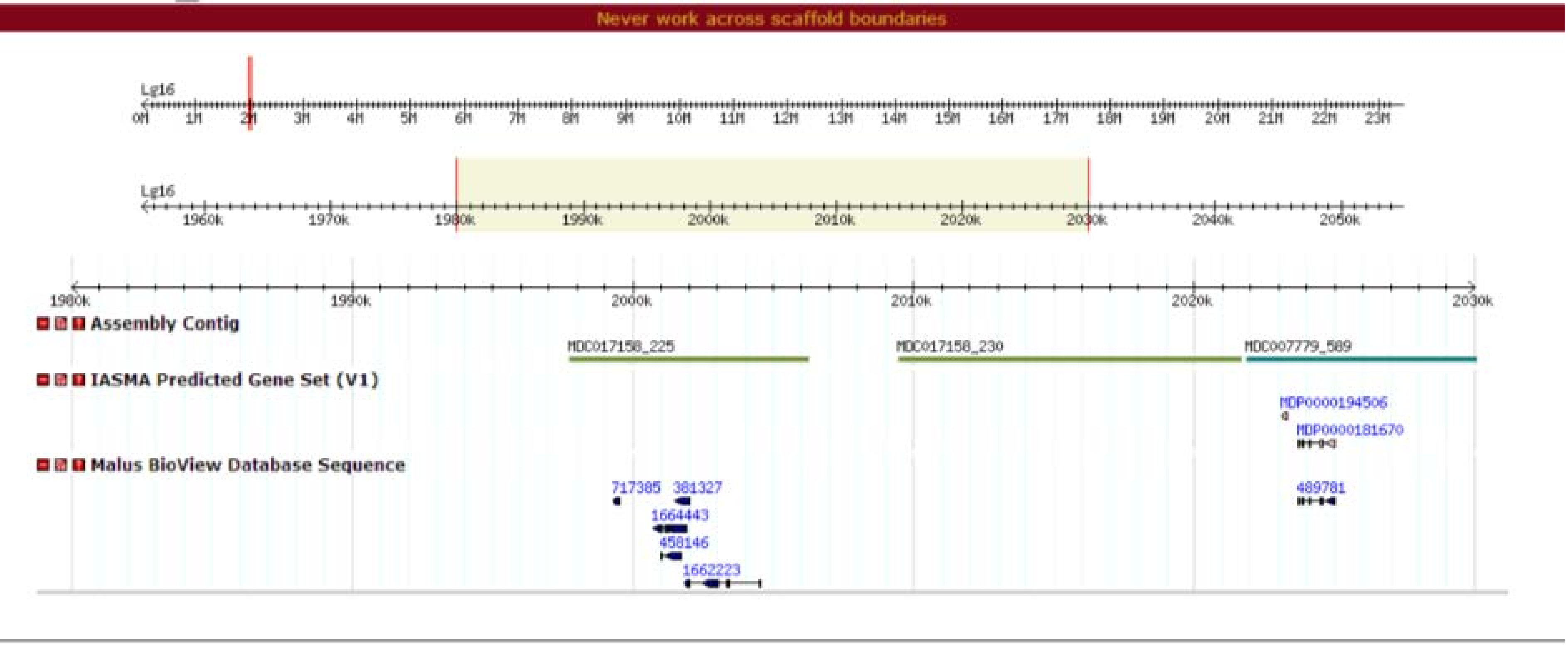

Cytoskeleton protein

D-aminoacyl-tRNA deacylase

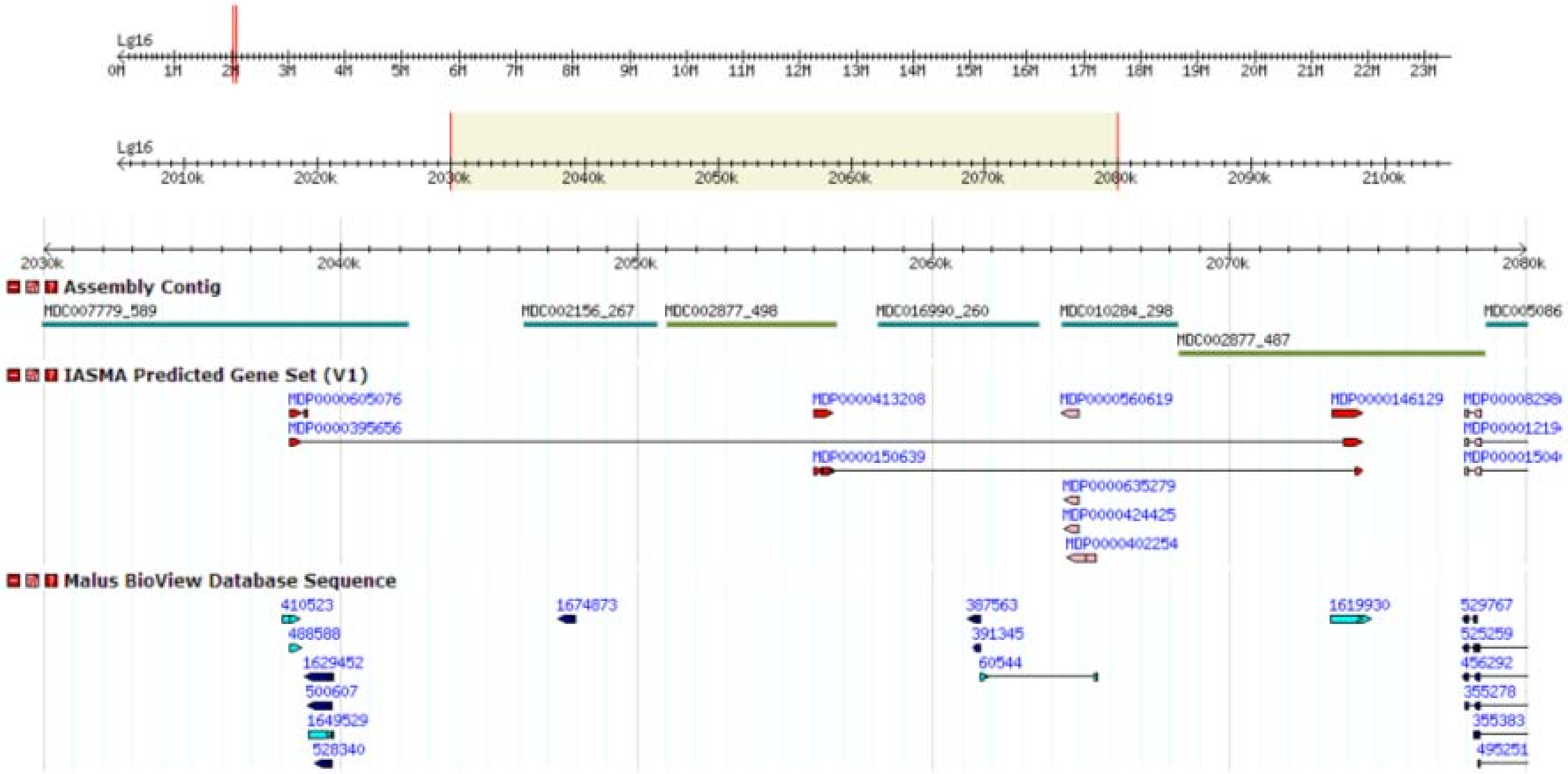

Flavonol sulfotransferase-like

D-aminoacyl-tRNA deacylase
